# Fabrication of Amorphous Silicon–Carbon Hybrid Films Using Single-Source Precursors

**DOI:** 10.1021/acs.inorgchem.3c01846

**Published:** 2023-09-13

**Authors:** Aileen Sauermoser, Thomas Lainer, Andreas Knoechl, Freskida Goni, Roland C. Fischer, Harald Fitzek, Martina Dienstleder, Christine Prietl, Anne-Marie Kelterer, Christine Bandl, Georg Jakopic, Gerald Kothleitner, Michael Haas

**Affiliations:** † Institute of Inorganic Chemistry, Graz University of Technology; Stremayrgasse 9/V, 8010 Graz, Austria; ‡ Graz Centre for Electron Microscopy (ZFE), Steyrergasse 17, 8010 Graz, Austria; § Institute for Sensors, Photonics and Manufacturing Technologies, Joanneum Research, Franz-Pichler-Straße 30, 8160 Weiz, Austria; ∥ Institute of Physical and Theoretical Chemistry, Graz University of Technology, Stremayrgasse 9/II, 8010 Graz, Austria; ⊥ Institute of Electron Microscopy and Nanoanalysis, Technische Universität Graz, Steyrergasse 17, 8010 Graz, Austria; # Institute of Chemistry of Polymeric Materials, Montanuniversität Leoben, Otto-Glöckelstrasse 2, A 8700 Leoben, Austria

## Abstract

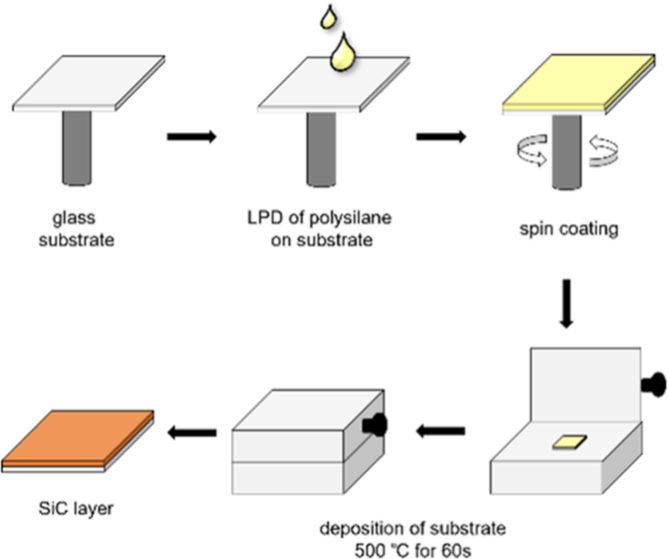

The aim of this study was the preparation of different amorphous silicon–carbon hybrid thin-layer materials according to the liquid phase deposition (LPD) process using single-source precursors. In our study, 2-methyl-2-silyltrisilane (methylisotetrasilane; **2**), 1,1,1-trimethyl-2,2-disilyltrisilane (trimethylsilylisotetrasilane; **3**), 2-phenyl-2-silyltrisilane (phenylisotetrasilane; **4**), and 1,1,2,2,4,4,5,5-octamethyl-3,3,6,6-tetrasilylcyclohexasilane (cyclohexasilane; **5**) were utilized as precursor materials and compared with the parent compound 2,2-disilyltrisilane (neopentasilane; **1**). Compounds **2**–**5** were successfully oligomerized at λ = 365 nm with catalytic amounts of the neopentasilane oligomer (**NPO**). These oligomeric mixtures (**NPO** and **6**–**9**) were used for the preparation of thin-layer materials. Optimum solution and spin coating conditions were investigated, and amorphous silicon–carbon films were obtained. All thin-layer materials were characterized via UV/vis spectroscopy, light microscopy, spectroscopic ellipsometry, XPS, SEM, and SEM/EDX. Our results show that the carbon content and especially the bandgap can be easily tuned using these single-source precursors via LPD.

## Abstract 2

Herein, the authors report on a variety of single-source precursors for Si/C thin film deposition. The so-obtained compounds were used for liquid phase deposition (LPD). The optimum conditions for the LPD process in the aspect of solution and spin coating parameters were developed. Subsequently, thin-layer materials were successfully deposited on 25 × 25 mm precleaned glass substrates at 500 °C. Finally, all thin-layer materials were characterized.

## Introduction

**Chart 1:**
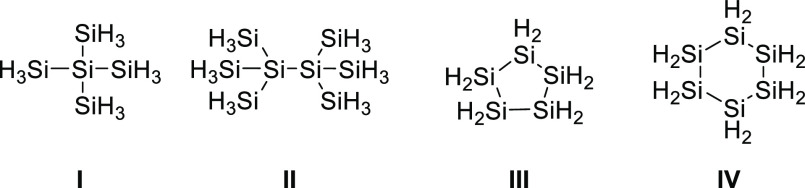
Currently Used Precursors for LPD Processing of Silicon Films

Recently, solution processing of silicon-based electronic devices has attracted much attention as an alternative to chemical vapor deposition (CVD), owing to the possibility of low-cost fabrication by printing processes. Moreover, it opens the possibility for large-area depositions and patterning materials.^1–5^ Recent studies in our laboratories and by others have demonstrated the principal feasibility of the liquid phase deposition (LPD) and processing of silicon films of satisfactory quality.^6–12^ Therefore, open chained and cyclic silicon hydrides such as compounds **I**–**IV** (Chart 1) were used as precursors and decomposed to elemental amorphous silicon upon heating to temperatures above 300 °C. To be valid for an industrial application, solution processing of silicon-based devices must allow continuous manufacturing of all circuit components by successive deposition and printing steps in the same environment. However, in order to perform LPD the precursor molecules need to be either liquid or soluble in inert solvents.

In this context, the deposition of silicon-heteroelement thin-layer structures is of great interest. Silicon carbide (SiC) or carbon-doped silicon has evolved from a high potential wide-bandgap semiconductor to a widely acknowledged material in power electronics.^13,14^ Here, carbon and other heteroelements are usually added to silicon to perform bandgap engineering. Since carbon provides a very wide bandgap compared to silicon, the bandgap width of the assembled thin SiC layer material can be varied by changing the concentration of carbon in the blend, thus the higher the carbon concentration the wider the bandgap.^15^ The difference in the emitter region of crystalline silicon and carbon-doped crystalline silicon layers can be seen by comparing the bandgaps of a heavily carbon-doped SiC layer (2.2–3.3 eV) to the bandgap of undoped silicon (1.12 eV).^16^ Since the difference is quite high, we assumed that bandgap engineering of amorphous silicon thin-layer materials can lead to the same tendency. Moreover, carbon-doped silicon is used as a wide-bandgap field effect transistor device and is more mechanically, thermally, and chemically stable compared to undoped silicon.^16^ So far, most of the known SiC layer materials have been prepared by alloying silicon with carbon and using remote plasma-enhanced CVD, whereas the main obstacle is the extremely low solubility of carbon in silicon.^17,18^ Again, the solution process can be seen as a promising alternative to the vapor methods. Nevertheless, the yield of such methods does not yet exceed that of vapor methods.^19–21^

To date, there are only a few reports on LPD-processed functional silicon layers in the literature. In those studies the use of more than one precursor raised considerable problems, thus using hydrosilanes as a precursor for the deposition of functional silicon films, which contain one or more heteroatoms covalently linked to silicon (single-source precursor), could offer the right solution.^22–25^ In many cases single-source precursors were shown to be ideal for producing thin films since they provide a simple route to these materials and therefore reduce the likelihood of side reactions and the associated formation of contamination of nonstoichiometric films of inferior quality.^26–28^ However, in comparison with carbon chemistry, the systematic functionalization of higher silicon hydrides for silicon-based single-source precursors has not been accomplished so far. Therefore, the synthesis of different single-source precursor materials with carbon as a heteroelement to achieve thin-layer materials via LPD and their characterization was the main target of this work.

## Results and Discussion

### Synthesis and Characterization of Precursor Molecules

**Scheme 1:**
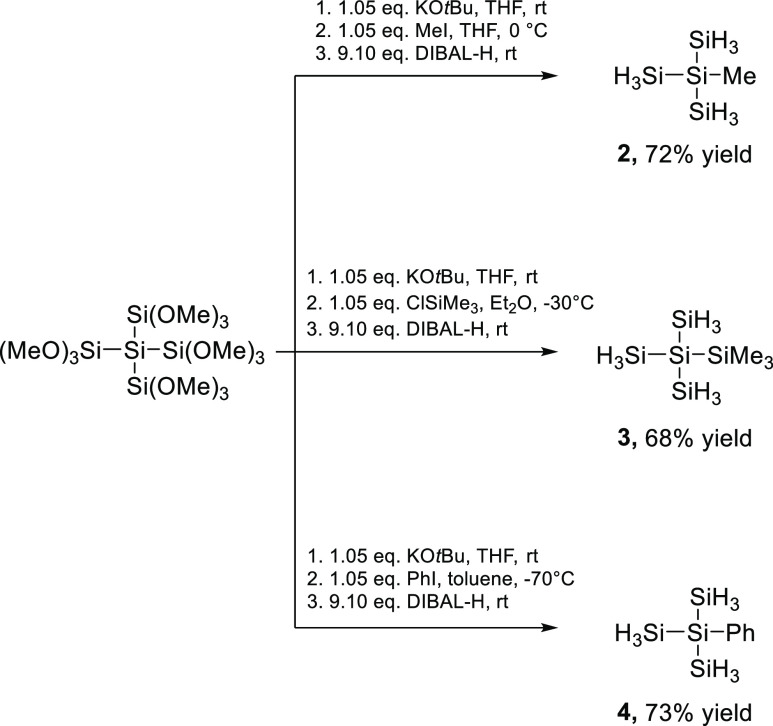
Synthesis of Precursor Molecules **2–4**

The branched precursor molecules **2**–**4** were synthesized using our previously reported method (Scheme 1).^29^ Therefore, dodecamethoxyneopentasilane was reacted with equimolar amounts of KO*t*Bu and the so-formed anion was in situ reacted with the respective electrophiles. These derivatives were isolated and subsequently reacted in a substance with 9 equiv of DIBAL-H. Finally, compounds **2**–**4** were isolated by vacuum condensation (see the Experimental Section and publication^29^ for details). Compounds **2** and **3** are already reported but never used as precursors for LPD approaches. Compound **4** has not been reported so far. Analytical data are consistent with the proposed structure, exhibiting one resonance line in the ^29^Si NMR spectrum for the three SiH_3_-groups at δ = −93.1 ppm and one signal for the quaternary silicon atom at δ = −90.0 ppm. Moreover, proton-coupled ^29^Si NMR spectrum of **4** allowed the determination of the coupling constants, which are in the range of previously reported perhydridosilanes (the H_3_Si-groups display a quartet of multiplets (^1^*J*_Si–H_ = 197.2 Hz, ^3^*J*_Si–H_ = 3.3 Hz); the quaternary silicon atom shows a multiplet (^2^*J*_Si–H_ = 5.0 Hz).^30^

**Scheme 2:**
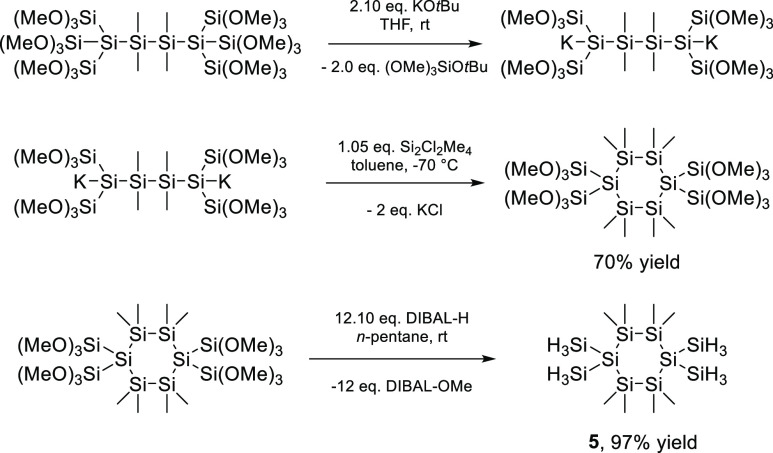
Synthesis of Precursor **5**

**Figure 1:**
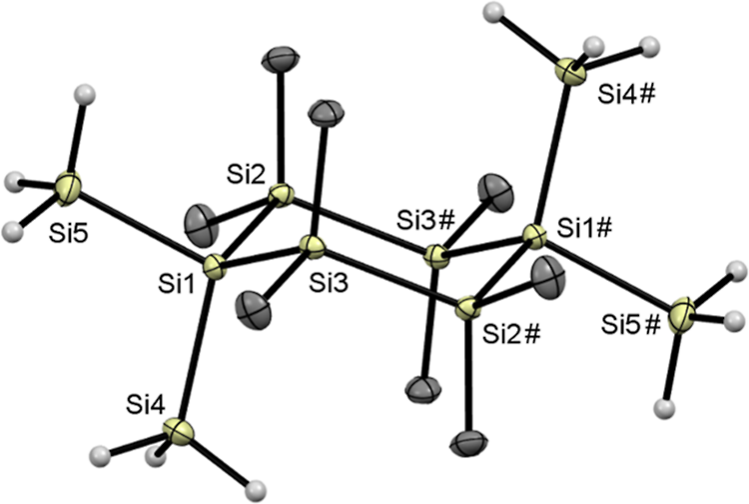
ORTEP representation of **5**. Thermal ellipsoids are drawn at the 50% probability level. Hydrogen atoms except for the hydrides are omitted. Selected bond lengths (Å) and bond angles (deg) with estimated standard deviations: Si(1)–Si(2) 2.3450(3), Si(1)–Si(3) 2.3473(3), Si(1)–Si(4) 2.3393(3), Si(1)–Si(5) 2.3348(3), Si(2)–Si(3) 2.3417(3), Si(2)–Si(1)–Si(3) 113.099(10), Si(4)–Si(1)–Si(2) 112.887(10), Si(4)–Si(1)–Si(3) 109.711(10), Si(5)–Si(1)–Si(2) 105.836(10).

As outlined in the introduction, cyclic precursors are also important LPD precursors. Consequently, we developed a synthetic approach toward the cyclic precursor **5** (see Scheme 2). Therefore, the branched hexasilane was reacted with 2 equiv of KO*t*Bu to the corresponding dianion. This dianion was in situ reacted with 1,2-dichlorotetramethyldisilane and the respective cyclohexasilane was obtained in good yields by crystallization. The cyclic hydrosilane was successfully formed in excellent yield (97%) using DIBAL-H to exchange all methoxy groups with hydrids. Again, analytical data is consistent with the proposed structure, exhibiting one resonance line in the ^29^Si NMR spectrum for the four SiMe_2_-groups at δ = −34.1 ppm, one signal for the four SiH_3_ groups at −97.1 ppm, and one signal for the quaternary silicon atom at δ = −146.8 ppm. The proton-coupled ^29^Si NMR spectrum of **5** allowed the determination of the coupling constants, which are also in the range of previously reported perhydridosilanes (the H_3_Si-groups display a quartet of multiplets (^1^*J*_Si–H_ = 193.53 Hz, ^3^*J*_Si–H_ = 4.3 Hz); the quaternary silicon atom shows a multiplet (^2^*J*_Si–H_ = 4.7 Hz). Finally, for compound **5**, we were able to grow crystals suitable for X-ray analysis, which were obtained by slowly evaporating a benzene solution at room temperature (see Figure 1). Compound **5** crystallized in the monoclinic space group *P*2_1_/*n* and the unit cell contains two molecules.

**Figure 2:**
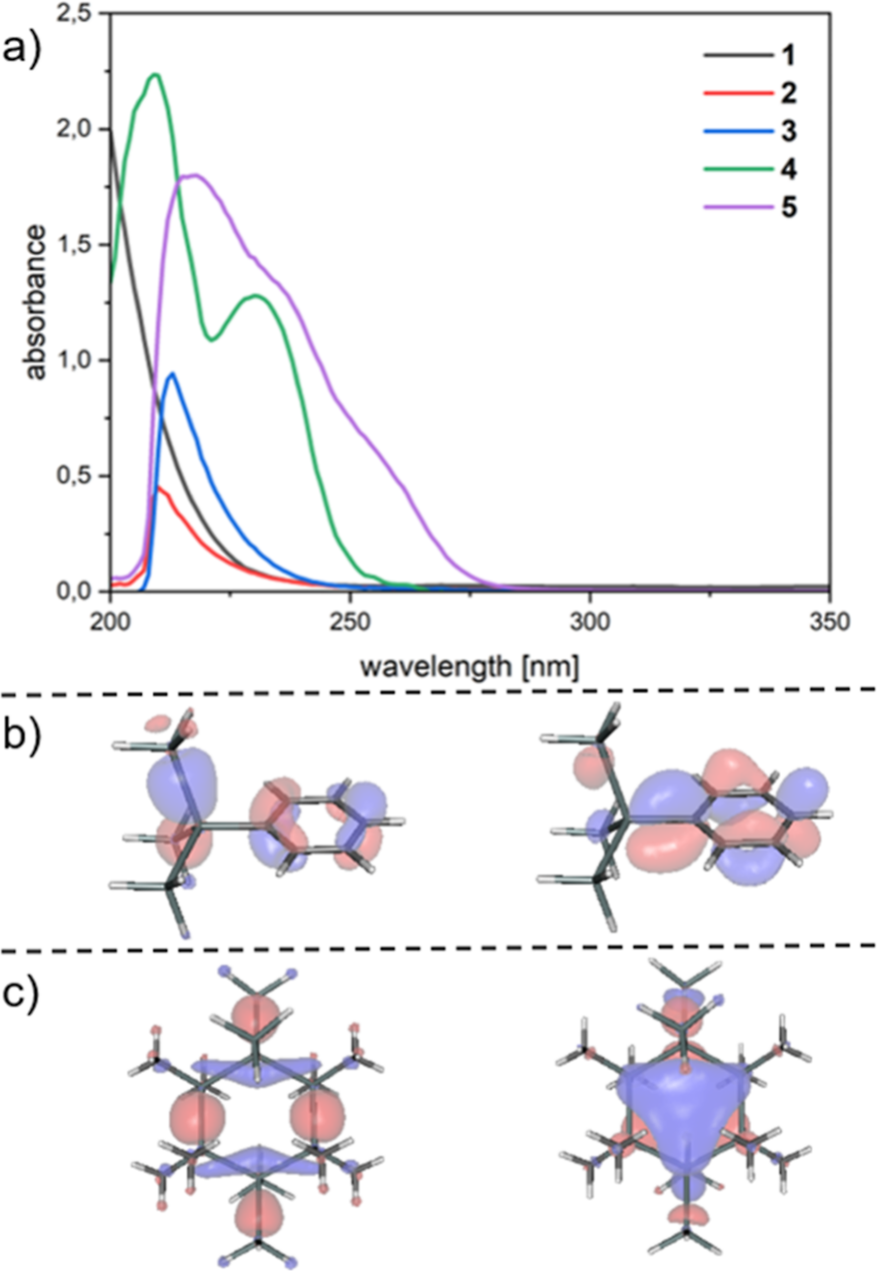
(a) UV/vis spectra of compounds **1–3** and **5** (*c* = 1 × 10^–4^ M; solvent = *n*-hexane) and of compound **4** (*c* = 1 × 10^–5^ M; solvent = *n*-hexane), (b) orbitals involved in the first transition for compound **4** (with a contour value of 0.04 au), and (c) orbitals involved in the first transition for compound **5** (with a contour value of 0.04 au).

As the LPD method requires a thermal or photochemical oligomerization of the precursor molecules, the UV/vis absorption spectra of **2**–**5** were measured and compared to those of parent compound **1** (Figure 2a). In order to determine the involved orbitals of the longest wavelength absorption excitation, we used DFT calculations and simulated the vertical excitations. A good qualitative agreement between experimental and calculated absorption maxima could be achieved. For all compounds except for compound **4**, the longest absorption band consists of σ–σ* excitations of the silicon skeleton from HOMO – 2, HOMO – 1, HOMO, and HOMO + 4 into LUMO, LUMO + 1, and LUMO + 2 orbitals with different mixing. On the other hand, for compound **4**, the first excitation consists of a π–π* transition from HOMO – 1 and HOMO into LUMO and LUMO + 1 (see the Supporting Information for details). As a representative example, the calculated Frontier Kohn–Sham orbitals for compounds **4** and **5** are depicted in Figure 2b,c.

### Oligomerization Experiments

**Scheme 3:**
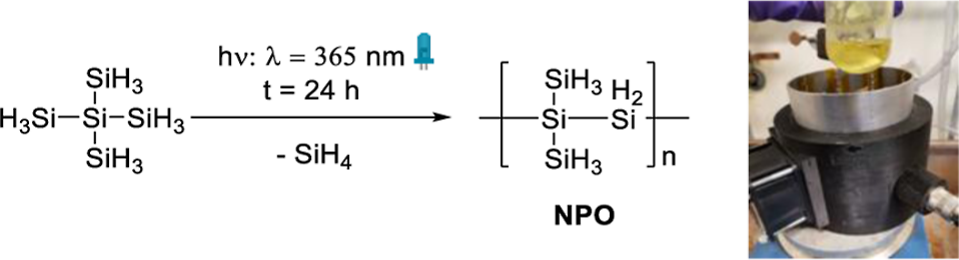
Oligomerization of Compound **1** at λ = 365 nm to Form NPO (Left); Photolysis Reactor Used for the Oligomerization at λ = 365 nm of Precursor Materials (Right)

In previous reports, the oligomerization process of branched hydropolysilanes was achievable only at a wavelength of λ = 254 nm in a quartz tube. The reason for this is based on the absorption properties of these derivatives.^7,9^ However, we were able to successfully achieve oligomerization for compound **1** in substance using a wavelength of λ = 365 nm in a nonquartz tube with our assembled reactor (see Scheme 3). See the Experimental Section and Supporting Information for details.

**Figure 3:**
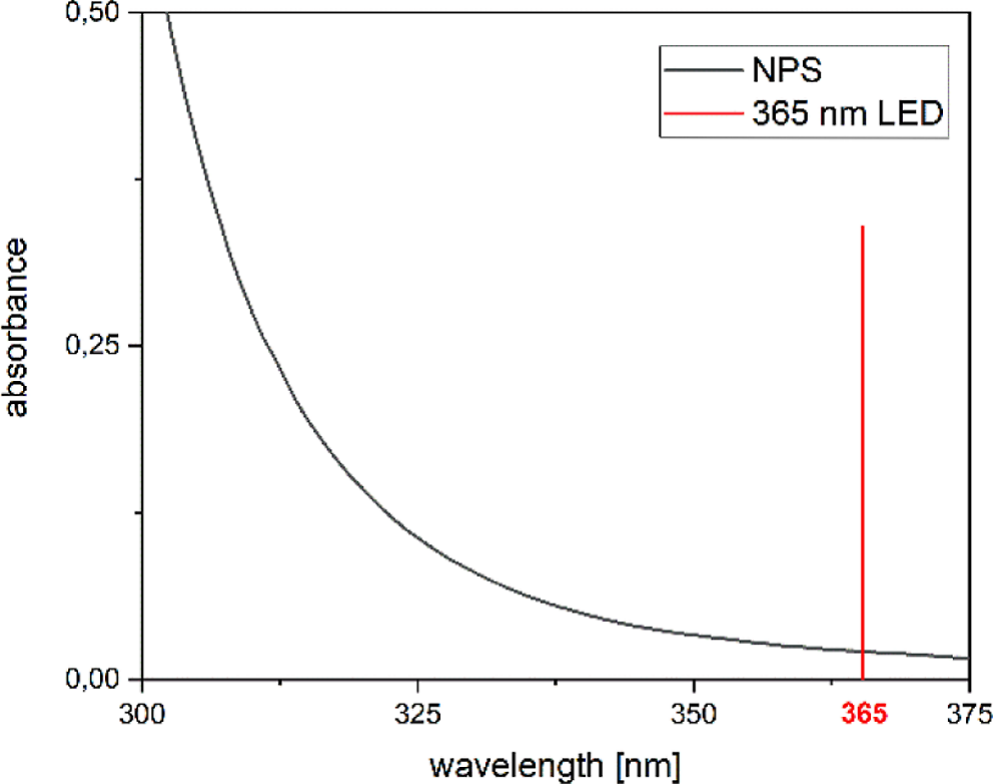
UV/vis spectra of compound **1** (neat).

Surprisingly, no absorption at this wavelength can be detected according to the UV/vis spectrum of **1**. Consequently, we recorded a UV/vis spectrum of **1** in substance and found a significant tailing of the absorption until 375 nm (Figure 3). The tailing is responsible for possible oligomerization at this wavelength. The photochemical oligomerization was stopped as soon as a solid polymer was precipitating out of the liquid phase, indicating an average molar mass of approximately 800–2000 g/mol.^7^ Encouraged by this finding, we wanted to adopt this method and irradiate our new single-source precursors at λ = 365 nm. For compounds **2**–**5**, the same conditions as for **1** were applied; however, no to very slow oligomerization was observed.

**Scheme 4:**
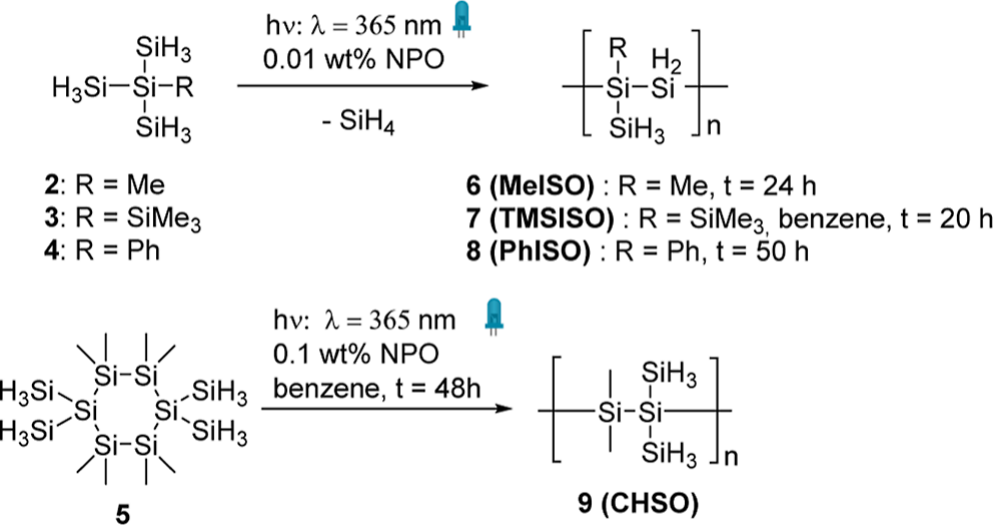
Oligomerization of Compound **2–5** at λ = 365 nm to Form the Oligomeric Mixtures **6–9**

For compound **2** we found a very slow oligomerization process (*h*ν at λ = 365 nm for 48 h shows nearly no oligomerization). Here, we assume that the low extinction coefficient is the main reason for the low photochemical activity. In addition, compounds **3** and **5** are solids, which were dissolved in benzene or *n*-pentane prior to irradiation. Again, no photochemically induced oligomerization was observed at λ = 365 nm. Looking at the orbitals involved in the longest wavelength absorption for compound **4**, we immediately realized that for this molecule the π–π* excitation is responsible for the inhibited photochemical oligomerization, as this band significantly overlaps the σ–σ* band. Consequently, we used our oligomerized neopentasilane (**NPO**) as an initiator to induce an oligomerization at this wavelength. On the basis of screening experiments, we found that 0.01 wt % of **NPO** is sufficient to induce a photochemical oligomerization for **2**–**4** and 0.1 wt % for precursor **5** (see Scheme 4). See the Experimental Section and Supporting Information for details.

**Figure 4:**
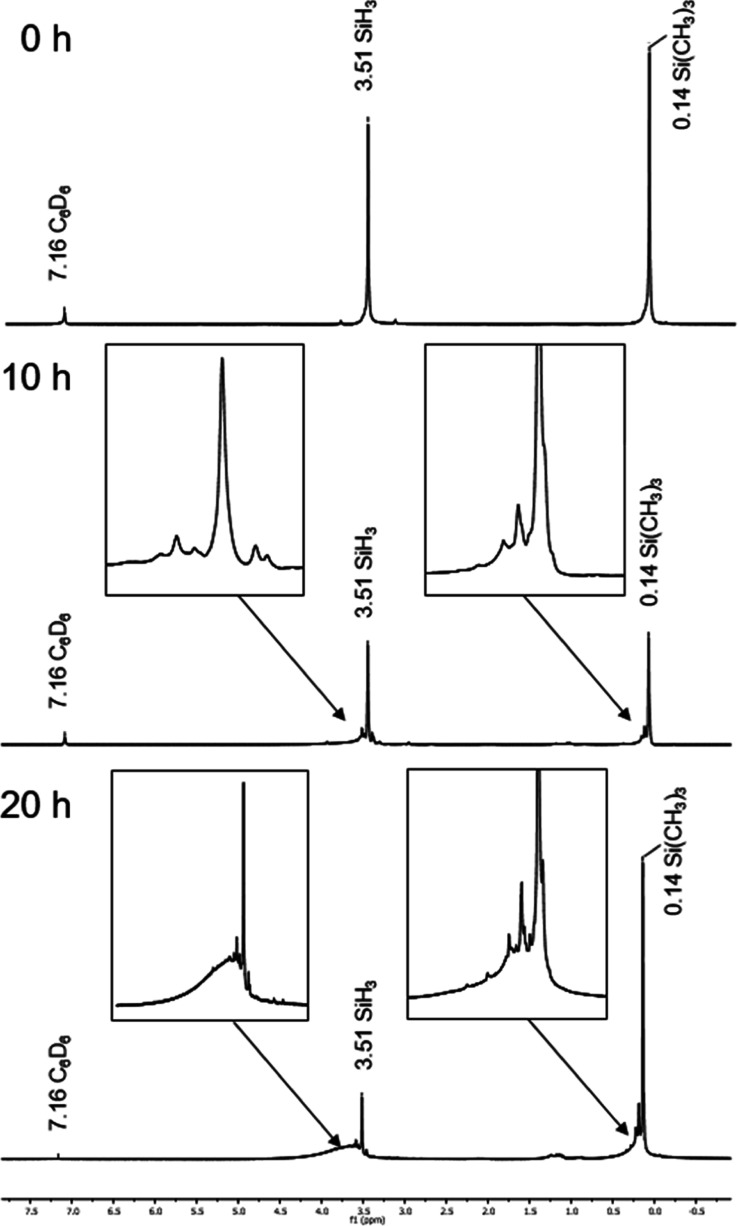
^1^H NMR spectra of the oligomerization process of compounds **3** to **7 (TMSISO)** (top: before photolysis at λ = 365 nm; middle: photolysis for 10 h at λ = 365 nm; bottom: photolysis for 20 h at λ = 365 nm).

The successful oligomerizations were characterized by ^1^H NMR spectroscopy. By comparison of the precursor materials to the oligomerized mixtures **6**–**9**, a significant broadening of the peaks was detected, which indicates the formation of oligomers. As an example, Figure 4 shows the formation of oligomeric mixture **7**. After 10 h of photolysis (λ = 365 nm), only slightly broader peaks were visible.

However, after 20 h of photolysis, two broad peaks right next to the signals of Si*H*_3_ and Si(C*H*_3_)_3_ were observed, which indicated the formation of the oligomeric mixture **7** (**TMSISO**). Another indicator for oligomerization of those compounds was the change in color after irradiation from a clear solution to a cloudy yellow color. For **8 (PhISO)** no specific broad peaks in the ^1^H NMR spectrum were detectable. In addition, we found various sharp signals in the Si–H region, indicating the formation of multiple photoproducts. Again, the color of **8** changed from a clear solution to a cloudy yellow color indicating oligomerization. Similar to **8**, no broad peaks in the ^1^H NMR spectrum were visible for **9 (CHSO)**. However, similar to other oligomeric mixtures (**6**–**8**), a change in color after irradiation from a clear solution to cloudy yellow was visible. It is important to mention that all of the obtained oligomeric mixtures still contain a significant amount of starting material (see Figures S12–S16). This is based on the necessity that the respective monomers serve also as solvents for the formed oligomers.

### Thin-Layer Materials

**Figure 5:**
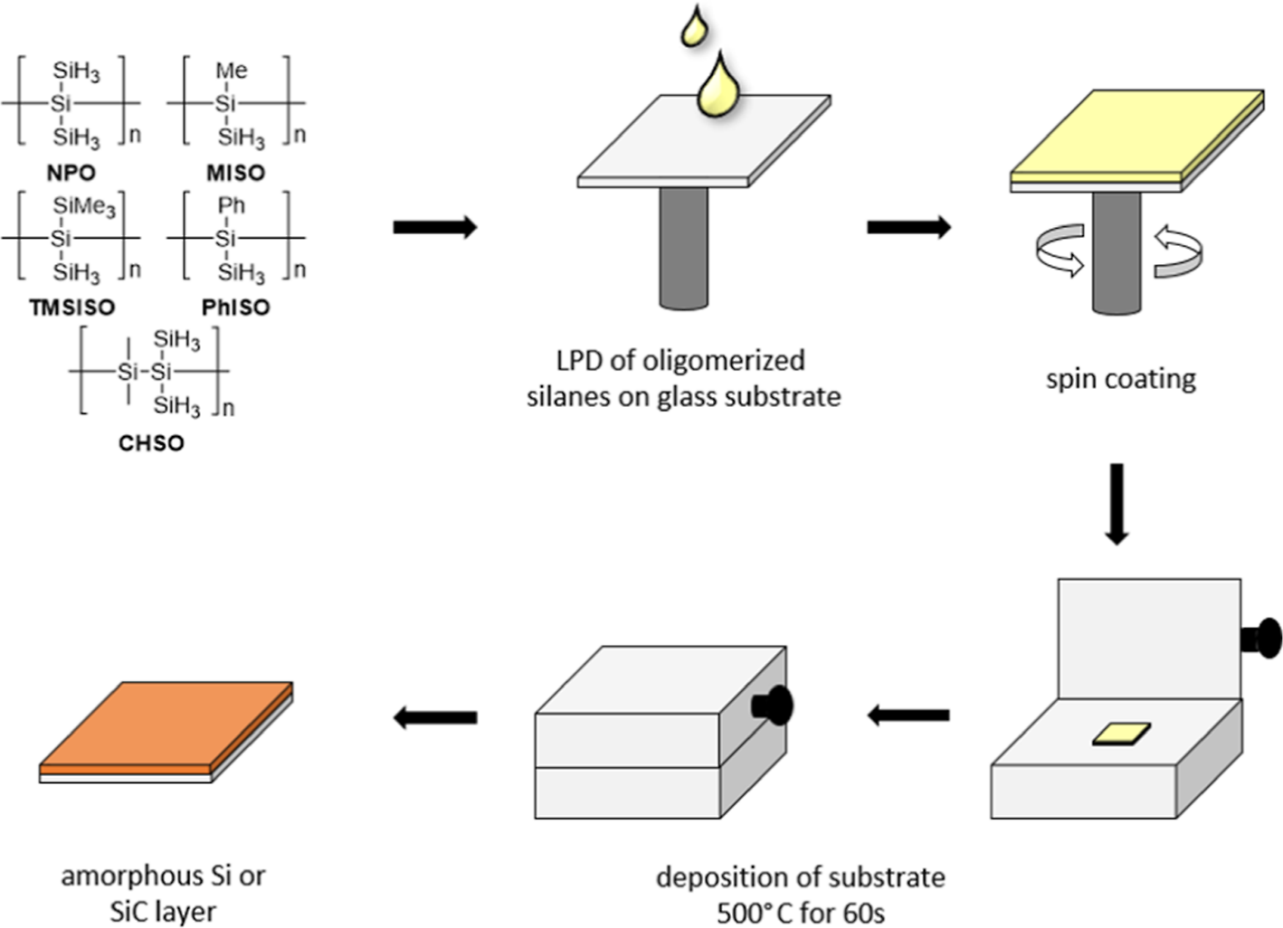
General procedure of the LPD process of oligomeric mixtures **6**–**9** and **NPO**.

With the successfully synthesized oligomeric mixtures **6**–**9** thin-layer materials were fabricated (Figure 5) and fully characterized.

Therefore, glass substrates were cut accordingly to fit the mask for the spin coating process (25 × 25 mm). Those substrates were cleaned with deionized water as well as acetone and isopropanol (ultrasonic bath for 1–2 h). Subsequently, the substrates were dried in an oven at 120 °C for 2 h and then transferred into the glovebox. All compounds were spin-coated on glass substrates in a nitrogen atmosphere. The first issue was the use of the right solvent. Oligomers **6**–**9** and **NPO** are soluble in tetrahydrofuran, toluene, as well as cyclooctane. To achieve optimum solvent conditions for the LPD process, **NPO** was used as a benchmark to ease the optimization process of all other compounds. In order to convert our hydrosilanes to amorphous silicon a temperature of at least 440 °C is needed. Consequently, we used 500 °C to ensure complete conversion.^7^ This thermal conversion was performed on a hot plate.

Table 1 summarizes the optimized conditions for the spin coating process for compounds **6**–**9**, whereas Table 2 shows the optimized LPD process conditions for each oligomeric mixture.

**Table 1:** Optimized Conditions for the Prepared Spin-Coating Solutions

	concentration [wt %]	solvents	temperature [°C]
**NPO**	50	toluene	RT
**6**	50	toluene	–30
**7**	50	40 wt % toluene + 10 wt % cyclooctane	–30
**8**	50	40 wt % toluene + 10 wt % cyclooctane	–30
**9**	15	THF	RT

**Table 2:** Optimized Spin Coating Parameters of the Different Solutions

	volume [mL]	speed [rpm]	time [s]	annealing temperature [°C]
**NPO**	0.20	9000	20	500
**6**	0.20	9000	20	500
**7**	0.24	3000	10	500
**8**	0.24	4000	10	500
**9**	0.24	4000	10	500

**Figure 6:**
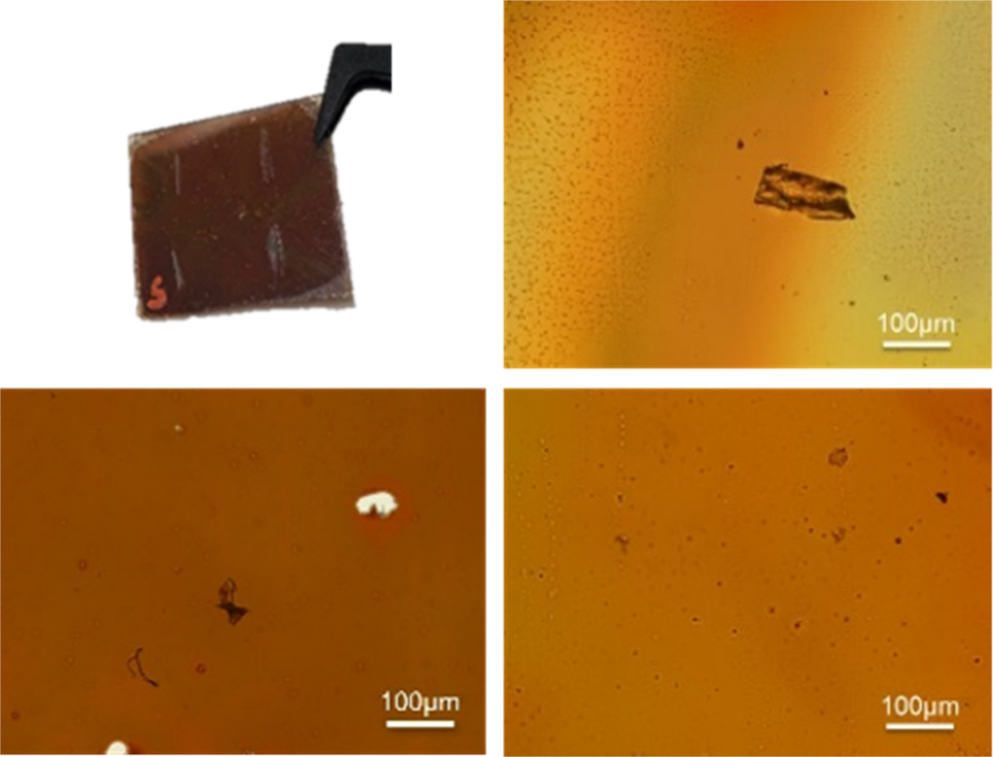
Picture of the successfully formed thin layer of 50 wt % **NPO** (scratches on the surface for analytical measurements) and light microscopy images at 200× magnification (top right: 30 wt % of **NPO** in toluene; bottom left: 50 wt % of **NPO** in 40 wt % toluene and 10 wt % cyclooctane; bottom right: 50 wt % of **NPO** in toluene).

Figure 6 shows the image of the thin layer itself as well as light microscopy images (200× magnification) of different solutions of **NPO**. As outlined in Figure 6, we observed the adsorption of small particles on the **NPO** layer. These particles can be attributed to our glovebox since the synthesis and oligomerization were also performed in this glovebox. As our study can be seen as a proof of concept, we can neglect these particles in later experiments.

**Figure 7:**
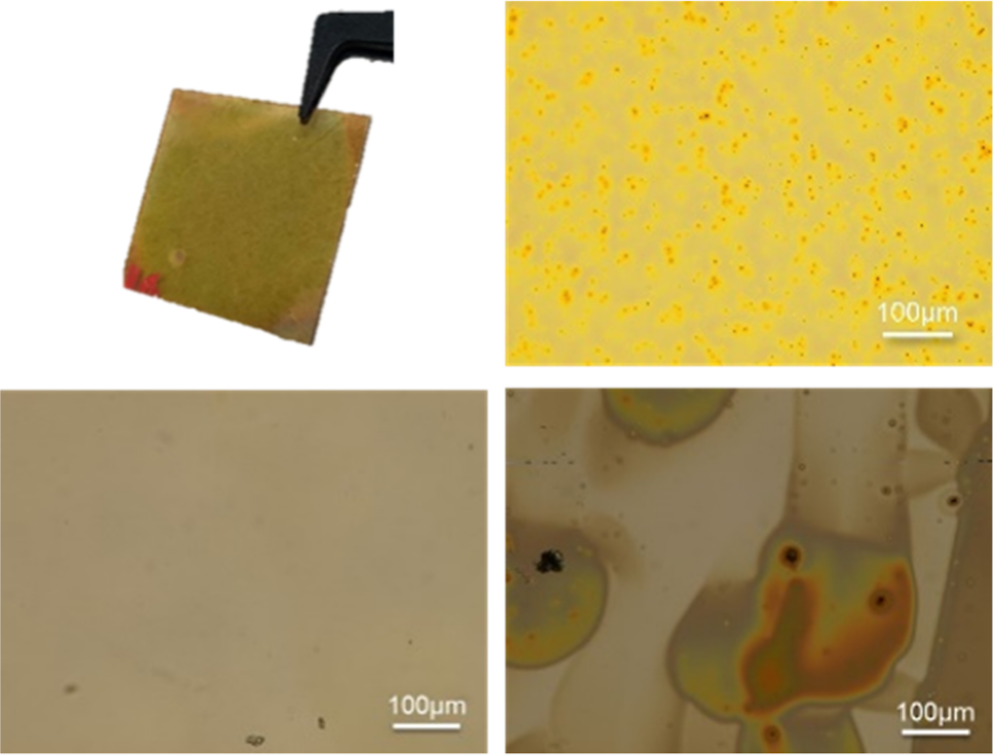
Picture of the successfully formed thin layer of 50 wt % **6** (**MeISO**) and light microscopy images of 200× magnification (top right: 50 wt % of **6** in toluene at −30 °C (high oligomeric/polymeric compounds not filtrated); bottom left: 50 wt % of **6** in toluene at −30 °C (filtrated); bottom right: 70 wt % of **6** in cyclooctane at −30 °C).

An additional step is required to achieve a high-quality thin-layer material with **6** as the precursor material. The solution of compound **6** had to be cooled to −30 °C before spin coating. Otherwise, no coating of the glass substrate was observable. The microscopic pictures offer information about the surface of each thin-layer material. Therefore, it was visible that the optimized conditions for **NPO** were applicable to **6 (MeISO**). However, due to the lower solubility of the oligomers of **6** in toluene, fine particles were observed via microscope. These particles were removed via filtration of the solution, and a thin SiC layer without holes, cracks, or particles was obtained (see Figure 7).

**Figure 8:**
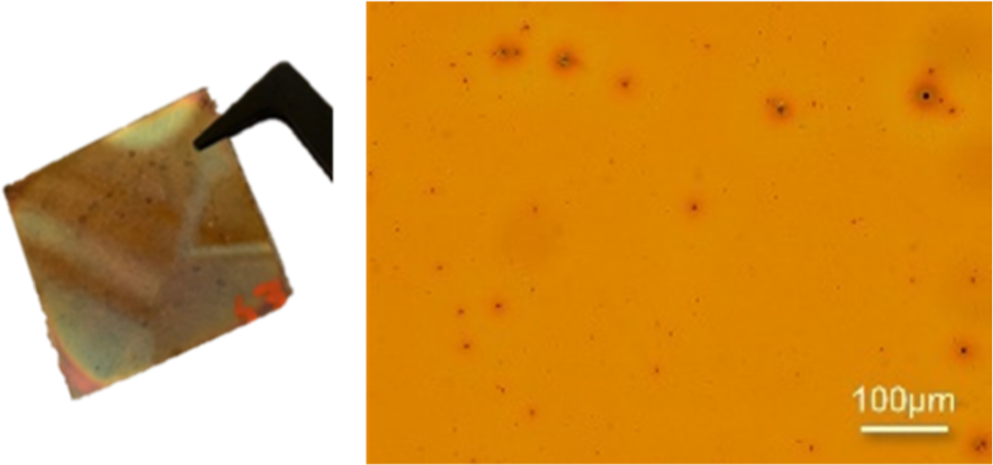
Picture of the successfully formed thin layer of 50 wt % **7 (TMSISO**) in 40 wt % toluene and 10 wt % cyclooctane and light microscopy images of the optimized SiC layer at 200× magnification.

Next, **7 (TMSISO**) was used to achieve thin SiC films. Again, we had solubility issues, which we were not able to solve with filtration, unlike for **6 (MeISO**), since the layer thickness was highly influenced by the filtration and also visible to the naked eye. Therefore, 10 wt % of cyclooctane was additionally added to the solution of 50 wt % of **7** in toluene. Moreover, the oligomer had to be cooled to −30 °C to obtain a thin layer on the glass substrate. In comparison to **NPO**, a thin layer of **7 (TMSISO**) was not achievable with the same spin coating parameters. In fact, the optimized solution of **7** needed much lower rotations per minute (3000 rpm) and a shorter spin coating time (10 s) to obtain a homogeneous surface layer. Figure 8 shows the obtained thin-layer material of **7 (TMSISO**).

**Figure 9:**
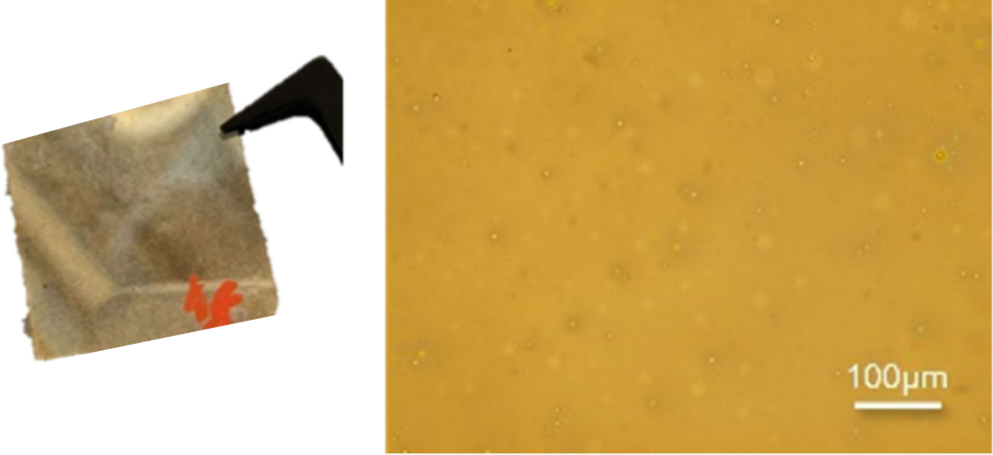
Picture of the successfully formed thin layer of 50 wt % **8 (PhISO**) in 40 wt % toluene and 10 wt % cyclooctane and light microscopy images of an optimized SiC layer at 200× magnification.

The same solvent and spin coating conditions of **7 (TMSISO**) were used for **8 (PhISO**). The only difference for those layer materials was the slightly higher rotations per minute (4000 rpm) compared to **7**. Figure 9 illustrates the surface in 200× magnification of the optimized thin layer of a solution of 50 wt % of **8** in 40 wt % toluene and 10 wt % cyclooctane (spin coating parameters: 4000 rpm; 10 s), as well as the taken picture of the successfully formed thin layer.

**Figure 10:**
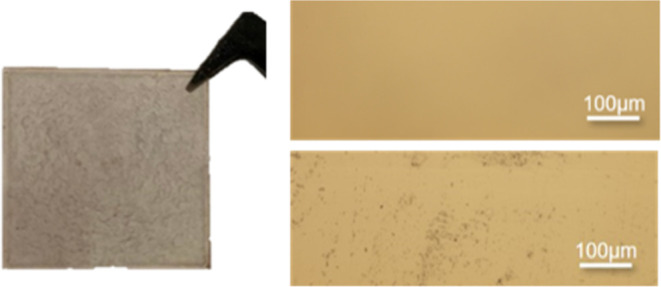
Picture of the formed layer of 15 wt % **9 (CHSO**) in THF and light microscopy images of 200× magnification (homogeneous (top right) and inhomogeneous (bottom right) parts of the thin-layer material).

The next target was the deposition of the oligomeric mixture **9** (**CHSO)**. For **9** we found that the solubility in our standard solvents (toluene, cyclooctane, and THF) is very low. We were only able to solve 15 wt % of **9** in THF and performed spin coating with this solution. Similar spin coating parameters as those for **7** were applied for **9**. The only difference was that spin coating at room temperature was possible. However, even at these low concentrations, we were not able to obtain homogeneous films. Therefore, microscopic images show the inhomogeneous surface of the thin-layer material, which was also visible without the help of a microscope (Figure 10).

**Figure 11:**
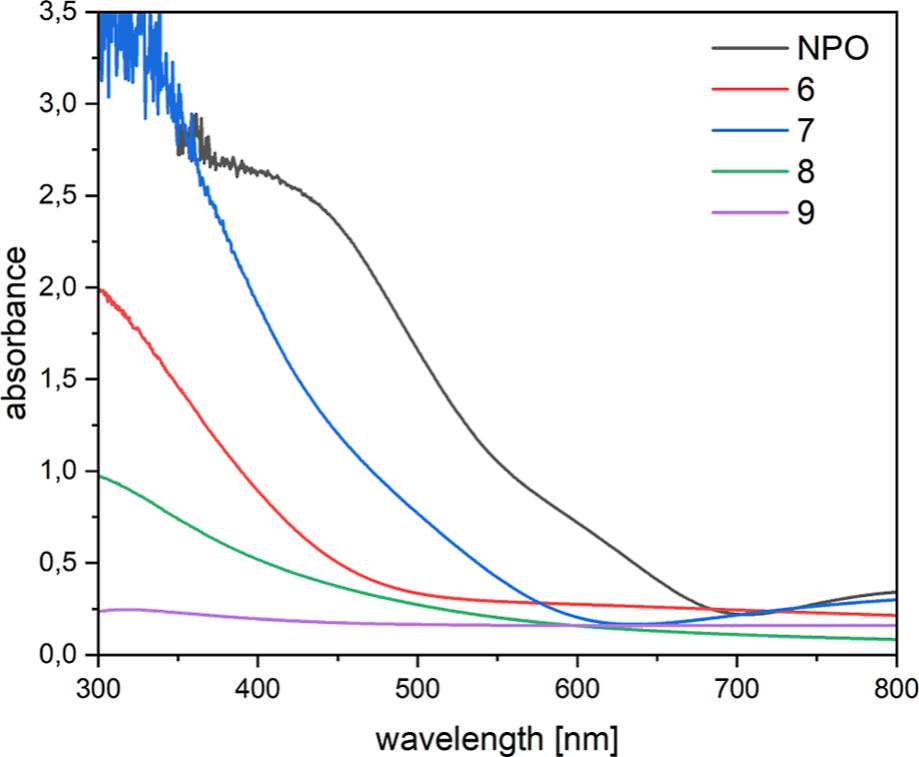
UV/vis spectra of the optimized thin-layer material of **NPO** and **6**–**9**.

The optical properties of the deposited thin-layer materials **NPO** and **6**–**9** were then further characterized. Therefore, UV/vis spectra were recorded. All layer materials show a similar trend. The absorptions took place in the range of 300–700 nm (Figure 11).

Compared to **NPO**, the other layer materials **6**–**9** show a less broad absorption band and no absorption in the region between 600 and 700 nm. Additionally, there was a trend in aspect-to-layer thickness detectable. The higher the absorbance of the thin-layer materials, the thicker the layer, which can be compared in the ellipsometry and scanning electron microscopy (SEM) part. By comparison of the absorption of the layer of material **9** to the other thin-layer materials (Figure 11), the absorbance is significantly lower (almost zero). This can be explained because of the low concentrations (15 wt % in THF) and the lack of solubility of **9**. Therefore, only very thin inhomogeneous layers were obtained and could not be further optimized.

From the absorption values shown above, the optical bandgap was determined, which can be seen in Table 3 (Tauc plots can be found in Figures S28–S30). The bandgap of **9** was not determined since the layer itself offered no homogeneous surface, and therefore, no high absorbance values could be detected.

**Table 3:** Obtained Optical Bandgap Values of NPO and **6–8**

	wavelength [nm]	optical bandgap [eV]
**NPO**	680	1.82
**6**	457	2.71
**7**	495	2.50
**8**	482	2.57

The bandgap of **NPO**-derived films was determined to be 1.82 eV, which is in the range of literature observed value for this material.^7^ All obtained values for the carbon-doped amorphous silicon layers are also in the range of literature-known optical bandgaps.^16^ The highest bandgap was obtained from thin layers of compound **6** (2.71 eV). Similar values for amorphous silicon films were found by Masuda, Shimoda, and co-workers by the usage of a two-component system.^23a^

**Figure 12:**
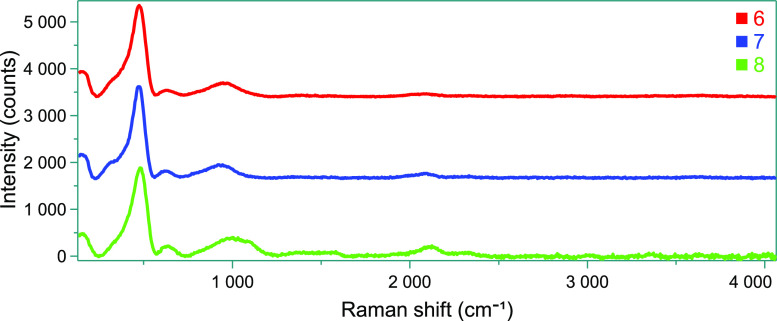
Raman spectra of the solution-processed a-Si/H films formed from **6** to **8**.

Furthermore, Raman spectroscopy was performed, which is the ideal method to determine the atomic arrangement of the silicon film. All films show the typical phonon bands of a-Si/H between 150 and 480 cm^–1^ (Figure 12). These spectra are comparable to the Raman spectra of vacuum-processed a-Si/H^31,32^ and amorphous silicon layers from cyclic silanes^11,33^ and branched silanes.^7,9^

**Figure 13:**
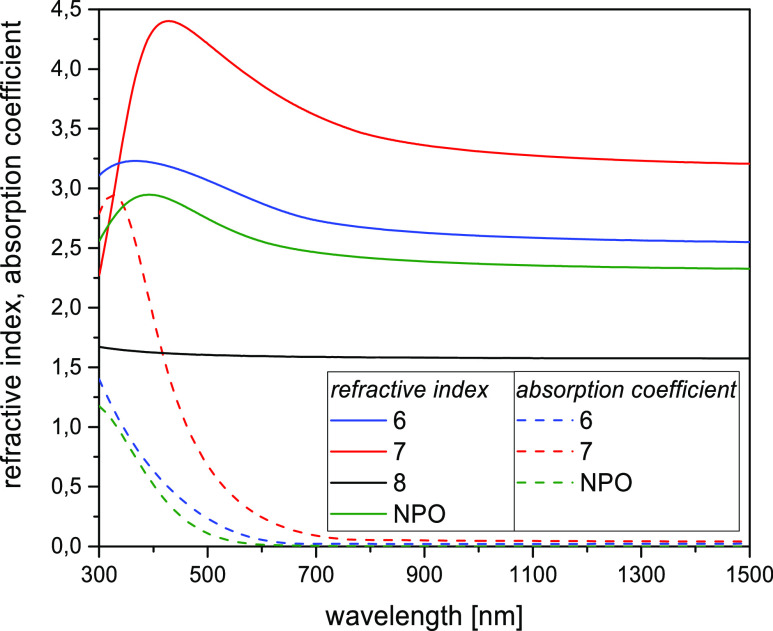
Optical constants *n* and *k* of the investigated thin films were determined by spectroscopic ellipsometry.

Additionally, the layer thickness is an important key parameter for the applicability. Therefore, we analyzed the layer thickness of all different optimized thin layers (**NPO** and **6**–**8**) via spectroscopic ellipsometry. A good fit of the ellipsometric parameters extrapolated with the model to the measured values was achieved for all four studied layers. Again, we used the thin film for **NPO** as a benchmark. The dielectric function or the optical constants of the investigated thin films were described with a model using a pole site in the UV outside the measured spectral range, a DC offset, a small (symmetric) Gaussian broadened, as well as a large (asymmetric) Cody–Lorentz oscillator in the UV/vis/NIR range. Moreover, a Drude term for the long-wavelength absorption in the IR was applied if necessary. The Cody–Lorentz formula typically is used for the description of the optical properties of pure amorphous silicon in the UV/vis range. Three of the thin films (**6**, **7**, and **NPO**) exhibit strong absorption extending from the UV into the visible region, coupled with the corresponding refractive index profile. It can be seen that these thin films are not completely optically homogeneous in the normal direction of the layer; adding a linear gradient of the optical constant into the model of about 10% could significantly improve the goodness of fit to the measured values. For **7** and **NPO** layers, the *n* and *k* values decrease from the top to the bottom of the layer, while for **6** they increase (see Figure 13).

These coatings also optically exhibit a low surface roughness of about 12 nm for **7** and **6** and about 3 nm for **NPO**. The ellipsometrically determined layer thicknesses are 73 and 70 nm for **7** and **6**, respectively, and distinctly larger −159 nm for **NPO**. The optical properties of compound **8** are significantly different. Here, no absorption can be detected within the ellipsometric sensitivity and the optical model gets by with one pole in the UV and a DC offset component. The layer thickness here is only about 31 nm, and the refractive index is much lower than that for the other three layers (see Table 4). Due to the small layer thickness, the statement about further nonidealities such as surface roughness and index gradients is not possible (rather, the simultaneous determination of thickness and refractive index must already be judged as clearly borderline). Further experimental details can be found in the Supporting Information (Figures S31–34).

**Table 4:** Measured Layer Thicknesses of the Layers Obtained for NPO and **6–8**^a^

	layer thickness [nm]	surface roughness [nm]
**NPO**	159	3
**6**	70 ± 2%	12
**7**	73 ± 8%	13
**8**	31	0

a For **6** and **7**, a thickness variation within the measuring spot could be determined.

**Figure 14:**
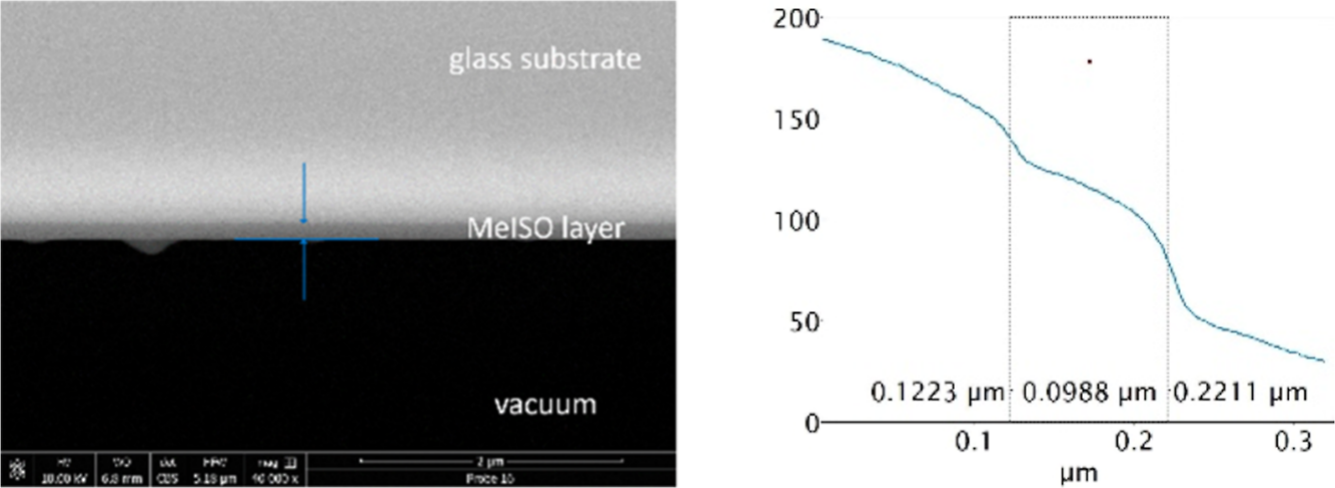
SEM images of MeISO (**6**) (left: 40,000× magnification; right: layer thickness measurement at 80,000× magnification).

In order to confirm the obtained thickness via ellipsometry, SEM was carried out for cross sections of thin layers of **6** and an overall thickness of approximately 100 nm was obtained (Figure 14). This speaks for the reliability of our spectroscopic ellipsometry measurements since a similar value was determined with SEM.

The layer thickness of compound **6** varies because of floating particles in the glovebox atmosphere as well as dust particles. To our delight, the overall thickness of this particular layer material offered values which we anticipated.

Additionally, since the determination of the layer thickness of all materials was successful and offered great value, the elemental composition of those compounds was also analyzed. This characterization method was achieved via SEM/EDX. The amount of oxygen was omitted since the layer materials were stored outside of the glovebox. (See Table S5 for the values of carbon, oxygen, and silicon). Additionally, small contaminations during the measurement of the elemental composition were also omitted. The measured and theoretical silicon and carbon contents can be found in Table 5. As expected, for thin-layer materials of **NPO** no carbon was detected, whereas the oligomeric mixtures **6**–**8**, offered carbon values. The amount of C varies in the different thin layers due to the different carbon groups of each starting material (**2**–**4**). When compared with the theoretical carbon content, a lower carbon content can be spotted for the thin-layer materials **7** and **8**. To our delight, we found that the carbon content for the different layers of **6** is almost identical to the theoretical value. We assume that during the oligomerization process, the incorporation of carbon in the amorphous silicon films of **6** is better than that in **7** and **8**. Microscopic images of the different layer materials at different magnifications can be seen in the Supporting Information (Figures S35–S39). Those images were obtained during the SEM/EDX measurement to further analyze the surface of each thin-layer material at higher magnitudes (200×–5.02k×) than for light microscopy (200× magnification). For the different thin-layer materials (**NPO** and **6**–**8**), homogeneous surfaces were detectable via SEM/EDX measurements. No holes, cracks, or other impurities were visible throughout the layer surfaces. Therefore, high-quality amorphous silicon layers of **NPO**, as well as amorphous silicon–carbon layers (**6**–**8**) were successfully deposited on glass substrates. In contrast to the thin layers of oligomeric mixtures **6**–**8**, no homogeneous surface layer for **9** was found. The oligomeric mixture **9** rather formed aggregates on the glass substrate after spin coating than an actual thin layer. This shows the cyclic compound **5**, can be used for oligomerization but is not suitable for LPD of thin-layer materials.

**Table 5:** Elemental Analysis of Three Different Layers of Oligomeric Mixtures **6–8** and NPO^a^

	measured			theoretical	
	C [wt %]	Si [wt %]		C [wt %]	Si [wt %]
**NPO**	0.00	100.00	**NPS**	0.00	100.00
	0.00	100.00	**2**	9.66	90.34
	0.00	100.00	**3**	20.42	79.58
**6**	9.92	90.07	**4**	39.08	60.92
	9.26	90.75			
	9.16	90.84			
**7**	6.10	93.90			
	5.68	94.33			
	5.26	94.74			
**8**	19.12	80.88			
	21.06	78.95			
	21.56	78.44			

a left: measured values by EDX; right: theoretical values.

In addition, we performed an elemental analysis of the oligomer mixtures before thermal treatment to back up our EDX measurements. Here we found that the carbon content has a similar tendency (see Table S7). Moreover, XPS was used to determine the surface composition of the layer. Again, similar Si–C ratios were found (see Table S6).

## Conclusions

In summary, we have successfully synthesized a variety of single-source precursors (**2**–**5**) for Si/C thin film deposition. Moreover, these compounds were photochemically oligomerized at λ = 365 nm. The five so-obtained oligomeric silicon mixtures (**6**–**9**) were used for LPD. The optimum conditions for the LPD process with respect to solution and spin coating parameters were developed. Subsequently, thin-layer materials of the oligomeric mixtures **6**–**8** were successfully deposited on 25 × 25 mm precleaned glass substrates at 500 °C. All thin-layer materials were characterized via light microscopy, SEM, SEM/EDX, spectroscopic ellipsometry, as well as UV/vis spectroscopy. Optical bandgaps of thin layers of compounds **6**–**8** were determined and compared to literature-known values for amorphous Si and SiC layers. Layer thicknesses of 31–73 nm were achieved. Elemental analyses were carried out and the obtained values were compared to theoretical ones. Therefore, thin amorphous silicon–carbon layers were successfully achieved. No homogeneous thin layer was obtained using the oligomeric mixture **9**. Further investigation into the possibility of the usage of solar cells will be done in the future.

## Experimental Section

### General Procedures

Caution! SiH_4_ is classified as an extremely flammable gas that spontaneously catches fire if exposed to air.

All experiments were performed under a nitrogen atmosphere using standard Schlenk techniques. Additionally, all thermal treatments were performed in a glovebox. Solvents were dried using a column solvent purification system.^34^ MeLi (1.6 M in Et_2_O), KO*t*Bu, and C_6_D_6_ (99.5 atom %, D) were used without any further purification. For the measurement of air-sensitive samples, C_6_D_6_ was additionally dried by 24 h reflux above a sodium/potassium alloy. Me(SiH_3_)_3_Si^35^ (**2**), Me_3_Si(SiH_3_)_3_Si^29^ (**3**), (Si(OMe)_3_)_4_Si,^36,37^ and K[(MeO)_3_Si]_2_Si(SiMe_2_)_2_Si[(MeO)_3_Si]_2_K^38^ were synthesized according to published procedures. Other commercial reagents were used as purchased, unless otherwise noted. ^1^H (200 MHz), ^13^C (50 MHz), and ^29^Si (40 MHz) NMR spectra were also recorded on a 200 MHz Bruker AVANCE DPX spectrometer in C_6_D_6_ solution (99.5 at. %, D) using the internal 2H-lock signal of the solvent. Additionally, infrared spectra were obtained on a Bruker Alpha-P Diamond ATR Spectrometer from the solid sample. Elemental analyses were carried out on a Hanau Vario Elementar EL apparatus. UV absorption spectra were recorded on a PerkinElmer Lambda 5 spectrometer. Photochemical experiments were run on a self-made photo reactor with arrays of various light-emitting diodes (LED) having an emission spectrum centered at 365, 405, 550, and 590 nm, respectively. The photoreactor setup comprises 24 LEDs per defined wavelength. Performed experiments were run with a total electrical output power of 25 W. Additionally, an integrated cooling system keeps a constant reaction temperature of 23 °C. The ellipsometric measurements were performed on a J. A. Woollam VASE Ellipsometer in the spectral range from 300 to 1500 nm with a step size of 10 nm and angles of incidence 65, 70, and 75°. The crystal suitable for single-crystal X-ray diffractometry was removed from a vial or Schlenk flask and immediately covered with a layer of silicone oil. A single crystal was selected, mounted on a glass rod on a copper pin, and placed in a cold N_2_ stream. XRD data collection for compound **5** was performed on a Bruker APEX II diffractometer with the use of an Incoatec microfocus sealed tube of Mo Kα radiation (λ = 0.71073 Å) and a CCD area detector. Table S1 in the Supporting Information contains crystallographic data and details of measurements and refinement for all compounds. Crystallographic data (excluding structure factors) have been deposited with the Cambridge Crystallographic Data Centre (CCDC) under the following numbers (**5**: 2262255).

### Synthesis of **2**

A solution of (Si(OMe)_3_)_3_SiK in 40 mL of THF was prepared from 3.00 g of Si(Si(OMe)_3_)_4_ (5.85 mmol) and 0.69 g of KO*t*Bu (6.14 mmol) and slowly added to a solution of 0.38 mL of MeI (6.14 mmol) in 40 mL of THF at 0 °C. The reaction mixture was stirred for 1 h at RT. The solvent was then removed via vacuum. 50 mL of *n*-pentane was added, and the remaining salts were filtered off. Recrystallization in *n*-pentane at −70 °C afforded 2.02 g of Me(Si(OMe)_3_)_3_Si (85%) as a white solid precipitate. As the next step, 8.06 mL of DIBAL-H (45.2 mmol) is slowly added to 2.02 g of MeSi(Si(OMe)_3_)_3_ (4.97 mmol) at 0 °C. The solution was allowed to stir at RT overnight. Recondensation at room temperature and 0.01 mbar afforded 0.49 g (72%) of **2** as a viscous liquid. ^1^H NMR (200 MHz, C_6_D_6_, ppm): δ 3.41 (s, 9H, Si*H*_3_), 0.17 (s, 3H, C*H*_3_). UV/vis measured in *n*-hexane (nm, [L mol^–1^ cm^–1^]): 210 [4517].

### Synthesis of **3**

A solution of (Si(OMe)_3_)_3_SiK in 40 mL of THF was prepared from 3.00 g of Si(Si(OMe)_3_)_4_ (5.85 mmol) and 0.69 g of KO*t*Bu (6.14 mmol) and slowly added to a solution of 0.78 mL of ClSiMe_3_ (6.14 mmol) in 40 mL of THF at −30 °C. The reaction mixture was stirred for 1 h at RT. The solvent was then removed via vacuum. 50 mL of *n*-pentane was added, and the remaining salts were filtered off. Recrystallization in *n*-pentane at −70 °C afforded 2.15 g of (SiMe_3_)Si(Si(OMe)_3_)_3_ (79%) as a white solid. As the next step, 7.50 mL of DIBAL-H (42.1 mmol) is slowly added to 2.15 g of (SiMe_3_)Si(Si(OMe)_3_)_3_ (4.63 mmol) at 0 °C. The solution was allowed to stir at RT overnight. Recondensation at 40 °C and 0.01 mbar afforded 0.61 g (68%) of **3** as a white solid. ^1^H NMR (200 MHz, C_6_D_6_, ppm): δ 3.52 (s, 9H, Si*H*_3_), 0.13 (s, 9H, C*H*_3_). UV/vis measured in *n*-hexane (nm, [L mol^–1^ cm^–1^]): 213 [9426].

### Synthesis of **4**

A solution of (Si(OMe)_3_)_3_SiK in 40 mL of THF was prepared from 3.00 g of Si(Si(OMe)_3_)_4_ (5.85 mmol) and 0.69 g of KO*t*Bu (6.14 mmol) and slowly added to a solution of 1.46 mL of PhI (6.14 mmol) in 40 mL of toluene at −70 °C. The reaction mixture was stirred for 1 h at RT. The solvent was then removed via vacuum. 50 mL of *n*-pentane were added and the remaining salts were filtered off. Again, the solvent was removed via vacuum, and recondensation at 180 °C and 0.01 mbar afforded 2.06 g of PhSi(Si(OMe)_3_)_3_ as a gelatinous oil (75%). As the next step, 7.13 mL of DIBAL-H (39.99 mmol) is slowly added to 2.06 g (4.39 mmol) of PhSi(Si(OMe)_3_)_3_ at 0 °C. The solution was allowed to stir at RT for another 3 h. Acidic aqueous workup was achieved by pouring the reaction mixture onto 120 mL of ice-cold H_2_SO_4_ (1 M, degassed) and allowing the suspension to meet RT while adding 60 mL of *n*-heptane under continuous stirring. The organic layer is dried over anhydrous Na_2_SO_4_, filtered off, and the solvent is removed under reduced pressure. Purification by fractional distillation at 0.01 mbar at 50 °C resulted in 0.64 g (73% yield) of a viscous colorless liquid (**4**). Anal. Calcd of C_6_H_14_Si_4_: C, 36.30%; H, 7.11%. Found: C, 36.41%; H, 7.25. ^1^H NMR (200 MHz, C_6_D_6_) δ 7.47 (m, 2H, aryl *H*), 7.05 (m, 3H, aryl *H*), 3.54 (s, 9H, Si*H*_3_). ^13^C NMR (50 MHz, C_6_D_6_, TMS, ppm): δ 136.3 (Aryl-*C*), 130.2 (Aryl-*C*), 129.6 (Aryl-*C*), 128.9 (Aryl-*C*). ^29^Si NMR (40 MHz, C_6_D_6_, TMS, ppm): δ −90.01 (*Si*(SiH_3_)_3_Ph); −93.1 (*Si*H_3_). UV/vis measured in *n*-hexane (nm, [L mol^–1^ cm^–1^]): 209 [223,180], 231, [127,700]. IR (*v* [cm^–1^]): 2129 (s, SiH).

### Synthesis of **5**

A solution of 2.45 g of K[(MeO)_3_Si]_2_Si(SiMe_2_)_2_Si[(MeO)_3_Si]_2_K (3.33 mmol) in 20 mL of THF was slowly added to a solution of 0.65 mL of SiCl_2_Me_4_ (3.50 mmol) in 20 mL of *n*-pentane at −70 °C. The mixture was stirred for an hour at RT. The solvent was removed via vacuum. 50 mL of *n*-pentane was added, and the salts were removed by filtration. A white solid precipitated in *n*-pentane at −70 °C to obtain 0.97 g of the 1,1,4,4-tetrakis(trimethoxysilyl)octamethylcyclohexasilane (70%). ^1^H NMR (200 MHz, C_6_D_6_, ppm): δ 3.59 (s, 36H, OC*H*_3_), 0.74 (s, 24H, C*H*_3_). ^29^Si NMR (40 MHz, C_6_D_6_, ppm): δ −33.8 (*Si*(CH_3_)_2_); −35.3 (*Si*(OCH_3_)_3_); −144.1 (*Si*(Si(OCH_3_)_3_)_2_).

2.70 mL DIBAL-H (2.70 mL, 15.2 mmol) is slowly added to 0.97 g of 1,1,4,4-tetrakis(trimethoxysilyl)octamethylcyclohexasilane (1.25 mmol) at 0 °C. The solution was allowed to stir at RT overnight. Acidic aqueous workup was achieved by pouring the reaction mixture onto 60 mL ice-cold H_2_SO_4_ (1 M, degassed) and allowing the suspension to warm up to RT while adding 30 mL of *n*-pentane under continuous stirring. The organic layer is dried over anhydrous Na_2_SO_4_, filtered off, and the solvent is removed under reduced pressure, resulting in 0.50 g of **5** (97%) as a white solid. Anal. Calcd of C_8_H_36_Si_10_: C, 23.25%; H, 8.78%; Si, 67.97%. Found: C, 23.39; H, 8.61%; Si, 68.00% ^1^H NMR (200 MHz, C_6_D_6_) δ 3.57 (s, 12H, Si*H*_3_), 0.31 (s, 24H, C*H*_3_). ^29^Si NMR (40 MHz, C_6_D_6_): δ −34.1 (*Si*(CH_3_)_2_), −97.1 (*Si*H_3_), −146.8 (*Si*(SiH_3_)_2_). IR (v [cm^–1^]): 2123 (s, SiH). UV/vis measured in *n*-hexane (nm, [L mol^–1^ cm^–1^]): 218 [17,970], 230 [14,421], 247 [8322].

### Photooligomerization of 2,2-Disilyltrisilane to NPO

For oligomerization, 0.41 g (6.56 mmol) of (SiH_3_)_4_Si was photolyzed at λ = 365 nm to form a yellow viscous liquid (**NPO**) after 24 h. ^1^H NMR (200 MHz. D_2_O capillary, ppm): δ 3.89–3.62 (Si*H*_3_).

### Photooligomerization of 2-Methyl-2-silyltrisilane (**2**) to **MeSIO** (**6**)

For oligomerization, 0.49 g of **2** (8.76 mmol) mixed with 0.01 wt % of **NPO** as initiator was photolyzed at λ = 365 nm to form a yellow viscous liquid (**6**) after 24 h. ^1^H NMR (200 MHz. C_6_D_6_, TMS, ppm): δ 3.65–3.33 (Si*H*_3_), 0.46 to −0.07 (C*H*_3_).

### Photooligomerization of 1,1,1-Trimethyl-2,2-disilyltrisilane (**3**) to **TMSISO** (**7**)

For oligomerization, 0.61 g of **3** (3.14 mmol) mixed with 0.01 wt % of **NPO** as an initiator in benzene was photolyzed at λ = 365 nm to form a milky yellow viscous liquid (**7**) after 20 h. ^1^H NMR (200 MHz, C_6_D_6_, ppm): δ 3.89–3.44 (Si*H*_3_); 0.29–0.11 (TMS).

### Photooligomerization of 2-Phenyl-2-silyltrisilane (**4**) to **PhISO** (**8**)

For oligomerization, 1.0 g (5.04 mmol) of **4** mixed with 0.01 wt % of **NPO** as initiator was photolyzed at λ = 365 nm to form a milky yellow viscous liquid (**8**) after 50 h. ^1^H NMR (200 MHz, C_6_D_6_, ppm): δ 7.52–7.39 (aryl *H*), 7.09–7.01 (m, aryl *H*), 3.62–3.35 (Si*H*_3_).

### Photooligomerization of 1,1,2,2,4,4,5,5-Octamethyl-3,3,6,6-tetrasilylcyclohexasilane (**5**) to **CHSO** (**9**)

For oligomerization, 0.5 g (1.2 mmol) of **5** mixed with 0.1 wt % of **NPO** as an initiator in benzene was photolyzed at 365 nm for 24 h. The solvent was removed under reduced pressure to form a yellow, viscous liquid (**9**). ^1^H NMR (200 MHz, C_6_D_6_, ppm): δ 3.58–3.33 (Si*H*_3_), 0.31 (C*H*_3_).

### Thin-Layer Materials

Thin-layer materials were spin-coated on glass substrates (25 mm × 25 mm). Prior to the spin coating, the substrates were cleaned with deionized water, acetone, and sonication in isopropanol (1–2 h). The spin coating speed for depositing the layers was varied from 3000 to 9000 rpm at 10–20 s. UV/vis spectroscopy was recorded with a Lambda 35 UV/vis-spectrometer from PerkinElmer (start wavelength: 900 nm; end wavelength: 300 nm; slit width: 1.0 nm; scan speed: 240 nm/min; data interval: 1.0 nm). The absorption spectra were measured in thin films on glass substrates after spin coating and thermal treatment at 500 °C. Images of the layer surface with a magnification of 200× were obtained by light microscopy. [Light Microscope BX60 from Olymp with an attached camera (Olymp)]. Layer Thickness was determined by FELMI-ZFE. The broad ion beam technique was used for the preparation of SEM (ESEM 450). Low vac mode was used to obtain images in different magnifications. The layers were measured in high-contrast CBS images using GMS3 software. Elemental analysis and further images of the surface layer were obtained via SEM/EDX. SEM micrographs and EDX spectra were collected using Tescan VEGA 3 SEM (Oxford Instruments plc, Abingdon, United Kingdom) with a tungsten source filament working at 20 kV. For SEM images, a resolution of 5 μm and a working distance of around 15 mm was used, and the EDX spectra were collected at a 0–10 keV scale. Prior to analysis, the samples were sputter-coated with gold.

## Supporting Information Available

NMR spectra for all precursor molecules and oligomerized molecules, characterization of the thin-layer material, and X-ray data of 5 and density functional theory computations (PDF)
Cartesian coordinates of calculated structures (XYZ)

## Supplementary Material

ic3c01846_si_001.pdf

ic3c01846_si_002.xyz
