# Supplementary material for: Fabrication of Amorphous Silicon–Carbon Hybrid Films Using Single-Source Precursors
Source: Inorg Chem. 2023 Sep 13;62(38):15490–501. doi: 10.1021/acs.inorgchem.3c01846 (PMC10523434; doi:10.1021/acs.inorgchem.3c01846)
Supplement: Supplementary file 1 — ic3c01846_si_001.pdf [file ic3c01846_si_001.pdf]

Supporting Information

Fabrication of Amorphous Silicon-Carbon Hybrid Films using Single Source Precursors

Aileen Sauermoser,[a] Thomas Lainer,[a] Andreas Knoechl,[a] Freskida Goni,[a] Roland C. Fischer,[a] Harald Fitzek,[b] Martina Dienstleder,[b] Christine Prietl,[c] Anne-Marie Kelterer,[d] Christine Bandl,[f] Georg Jakopic,[c] Gerald Kothleitner,[e] Michael Haas[a]*

[a] Institute of Inorganic Chemistry, Graz University of Technology; Stremayrgasse 9/V, 8010 Graz (Austria) E-mail: michael.haas@tugraz.at

[b] Graz Centre for Electron Microscopy (ZFE); Steyrergasse 17, 8010 Graz (Austria).

[c] Institute for Sensors, Photonics and Manufacturing Technologies, Joanneum Research Forschungsgesellschaft mbH, Franz-Pichler-Straße 30, 8160 Weiz (Austria)

[d] Institute of Electron Microscopy and Nanoanalysis, Technische Universität Graz, Steyrergasse 17, 8010 Graz (Austria).

[e] Institute of Electron Microscopy and Nanoanalysis, Technische Universität Graz, Steyrergasse 17, 8010 Graz (Austria).

[f] Institute of Chemistry of Polymeric Materials, Montanuniversität Leoben, Otto-Glöckelstrasse 2, A 8700 Leoben (Austria)

Table of Content

Analytical Section 3
NMR Spectroscopy 3
Precursor Molecules 3
Oligomerized Molecules 14
UV/Vis spectrometry – Precursor Molecules 19
Thin Layer Materials 23
Deposition and Solution Parameters 23
Optical Properties 23
Layer Thickness and Elemental Composition 27
References 34

Analytical Section

NMR Spectroscopy

Precursor Molecules

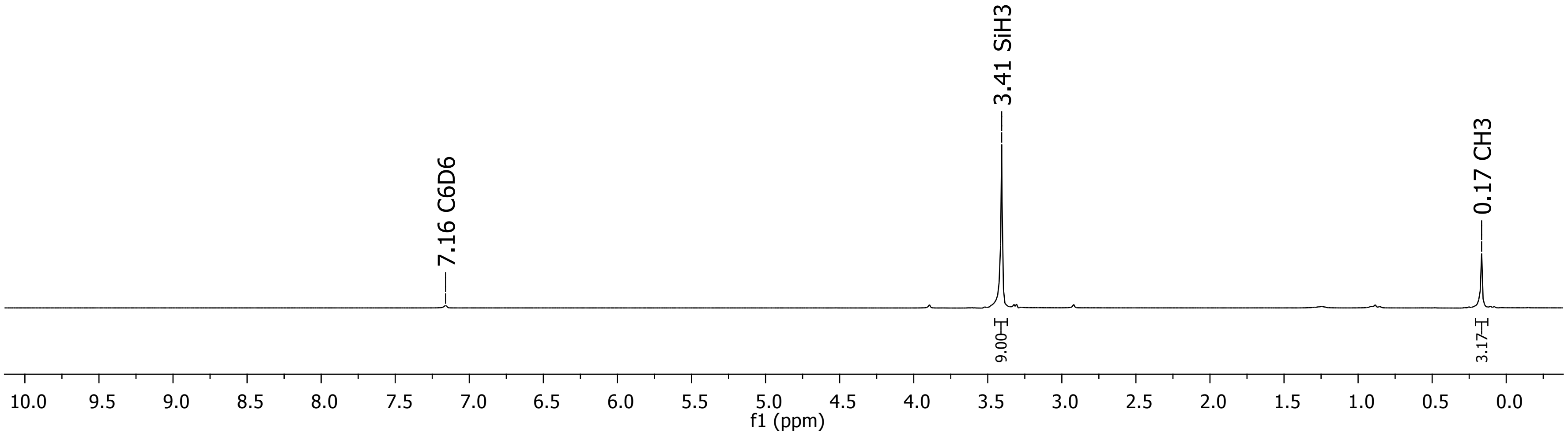

Figure S1. ^{1}H NMR spectrum of 2-methyl-2-silyltrisilane (**2**) (C_6D_6 solution, RT, ppm, 200 MHz)

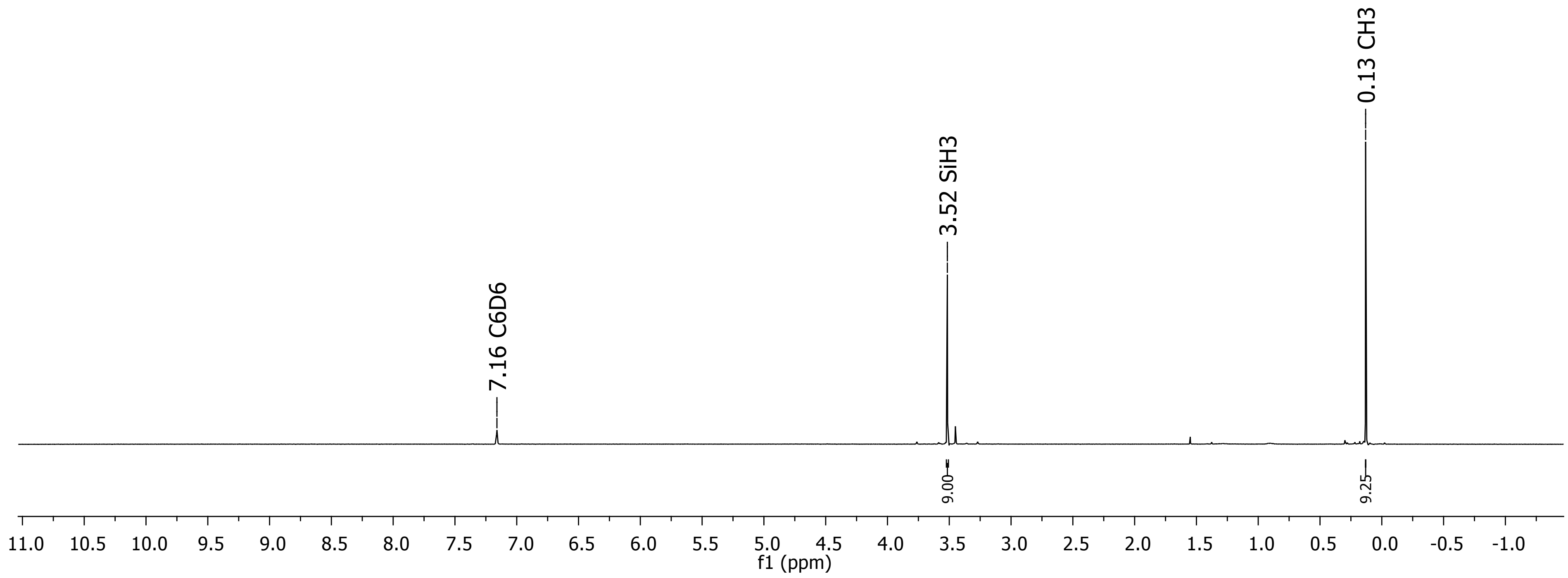

Figure S2. ^{1}H NMR spectrum of 1,1,1-trimethyl-2,2-disilyltrisilane (**3**) (C_6D_6 solution, RT, ppm, 400 MHz)

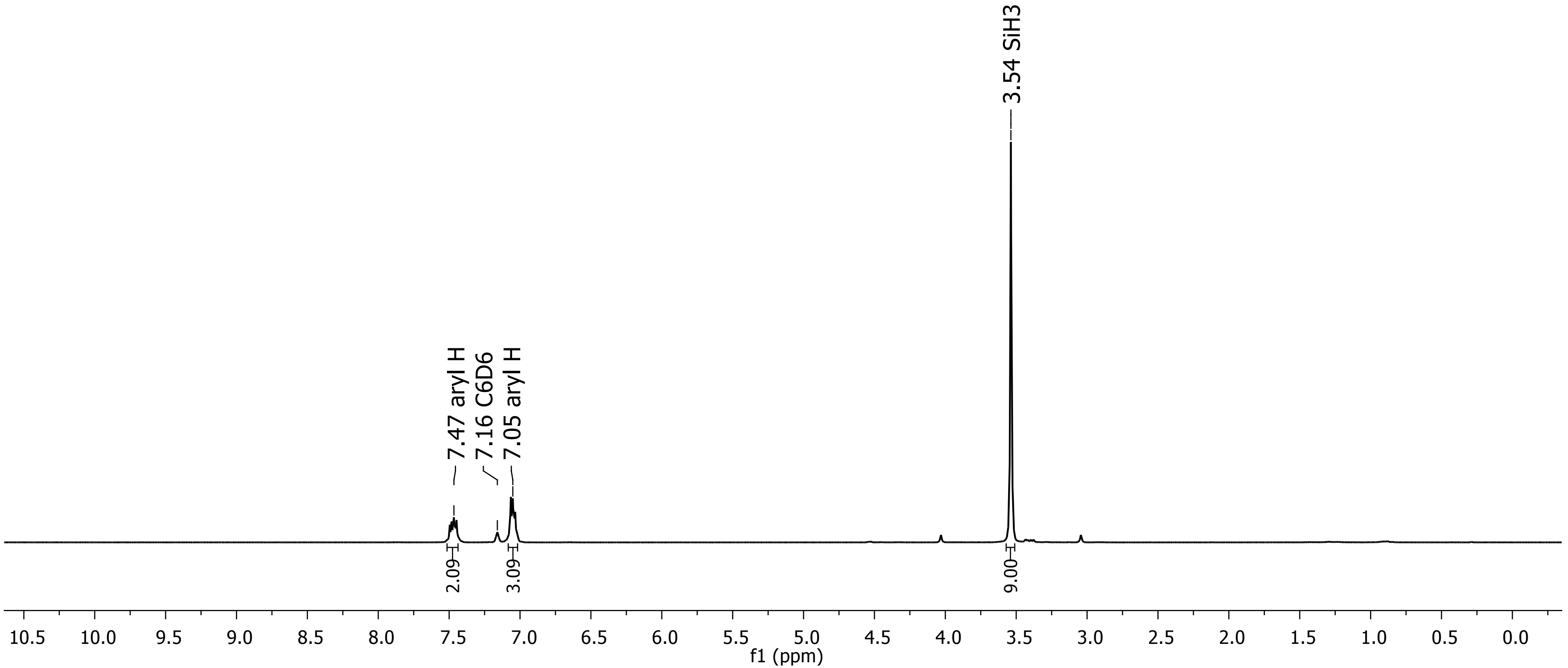

Figure S3. ^{1}H NMR spectrum of 2-phenyl-2-silyltrisilane (**4**) (C_6D_6 solution, RT, ppm, 200 MHz)

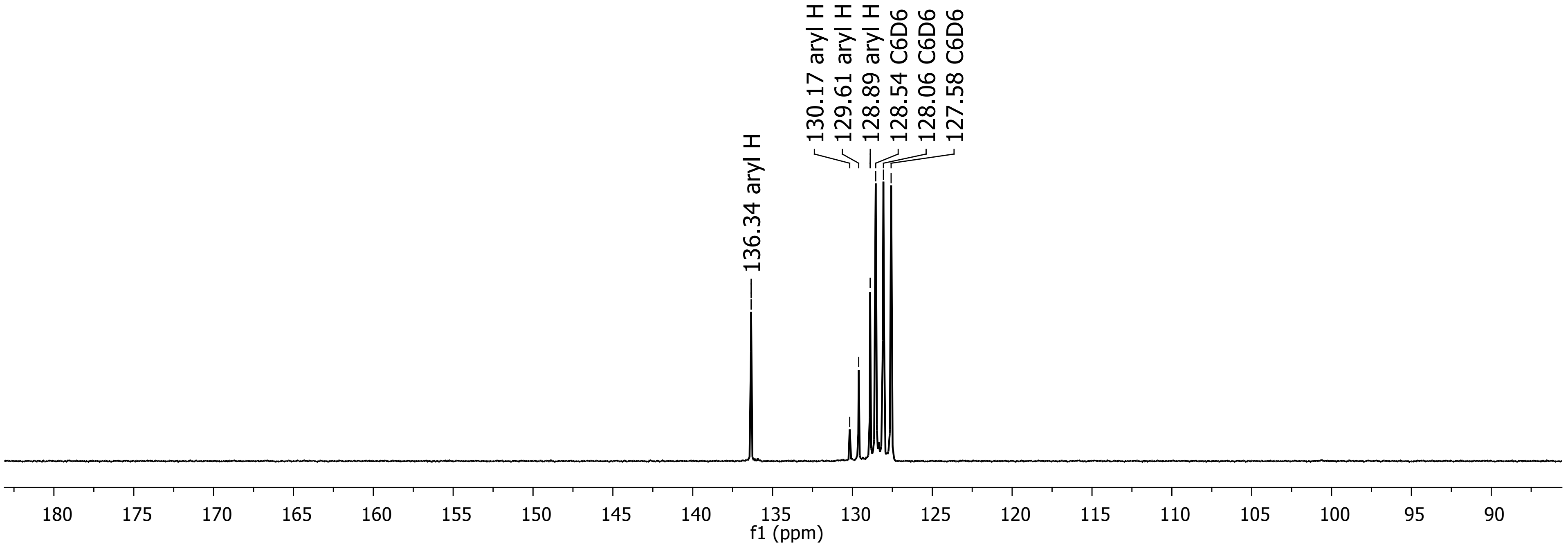

Figure S4. ^{13}C NMR spectrum of 2-phenyl-2-silyltrisilane (**4**) (C_6D_6 solution, 50 MHz, RT, ppm)

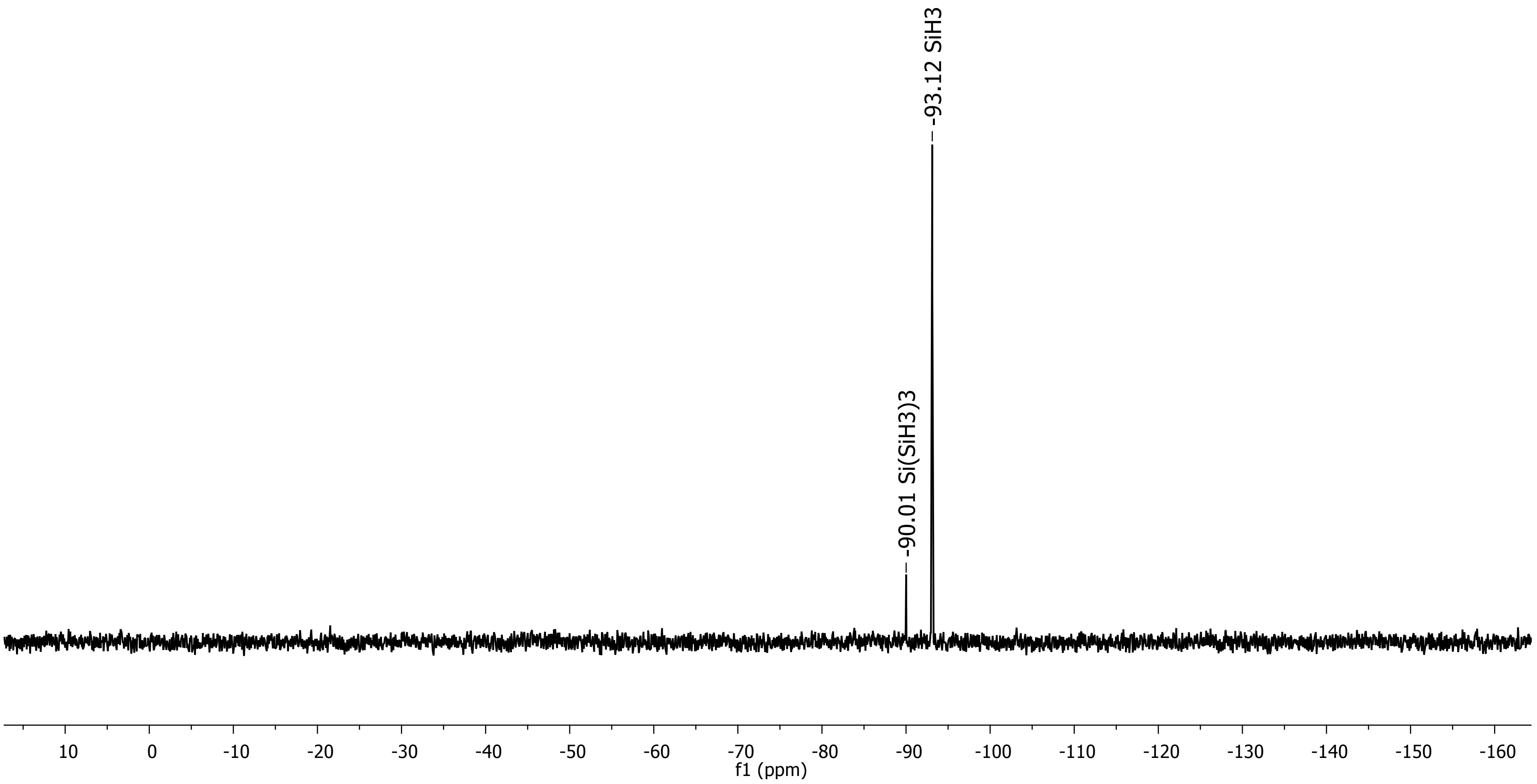

Figure S5. ^{29}Si-INEPT NMR spectrum of 2-phenyl-2-silyltrisilane (**4**) (C_6D_6 solution, 40 MHz, RT, ppm)

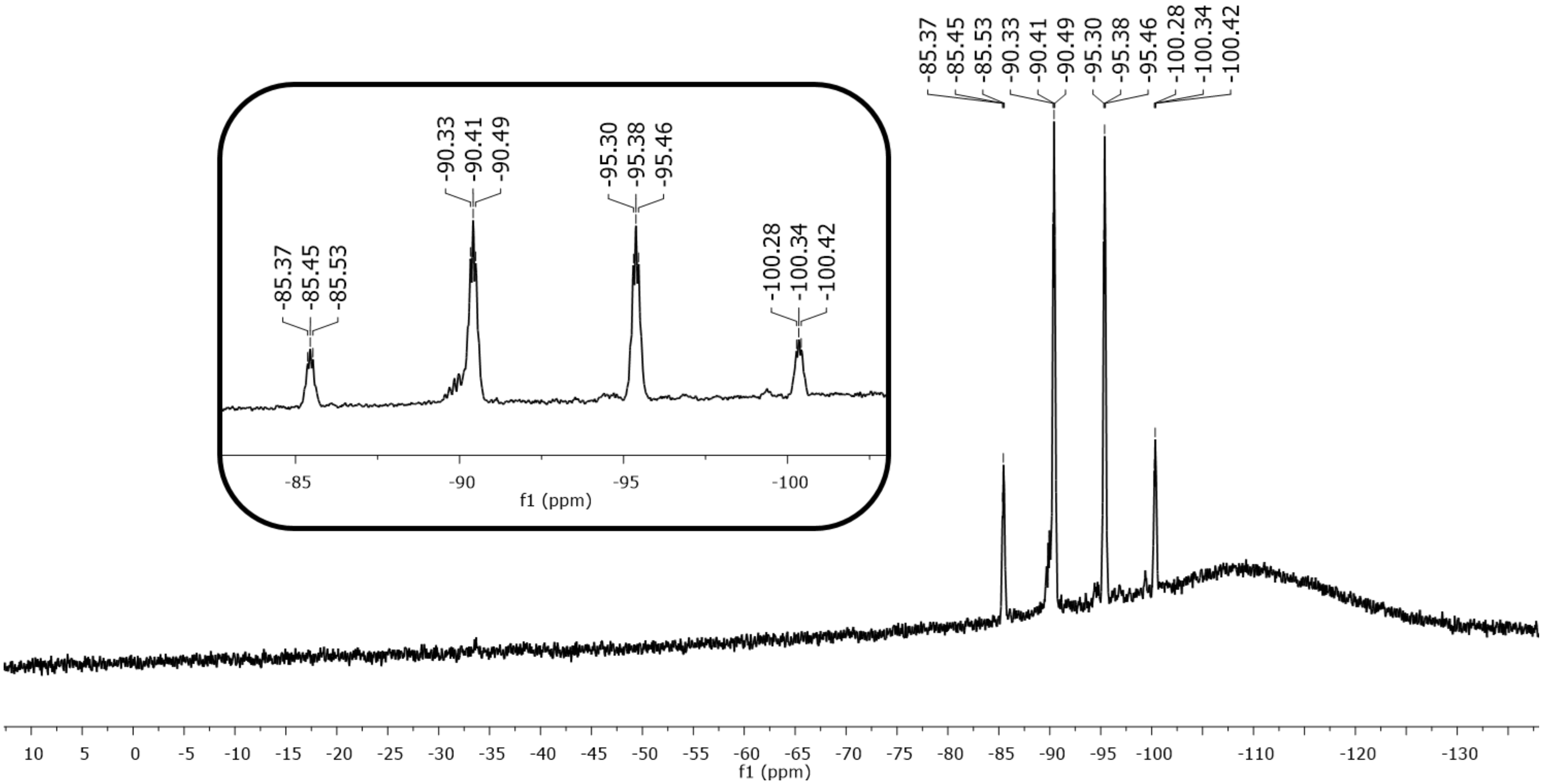

Figure S6. Proton-coupled ^{29}Si NMR spectrum of 2-phenyl-2-silyltrisilane (**4**) (C_6D_6 solution, 40 MHz, RT, ppm)

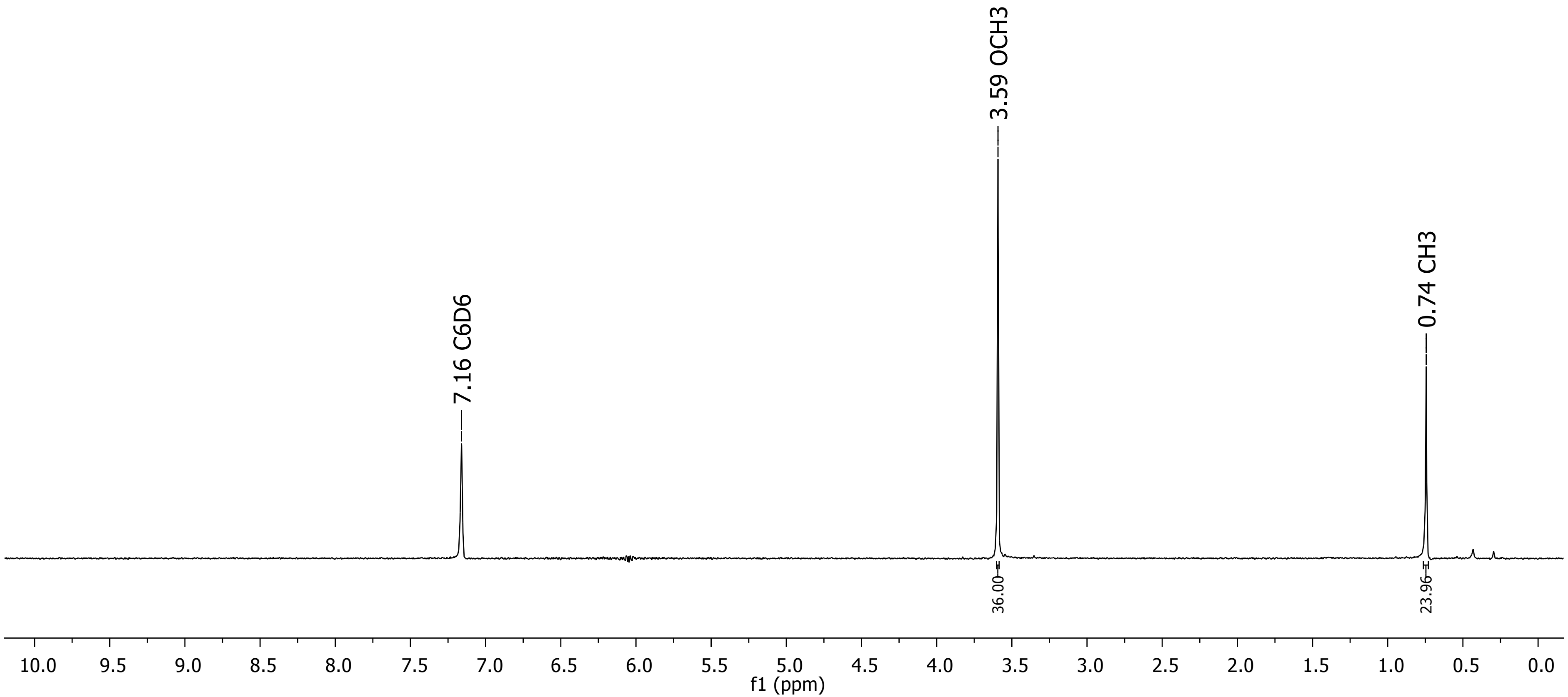

Figure S7. ^{1}H NMR spectrum of 2,2,3,3,5,5,6,6-octamethyl-1,1,4,4-tetrakis(trimethoxysilyl)cyclohexasilane (**5**) (C_6D_6 solution, 200 MHz, RT, ppm)

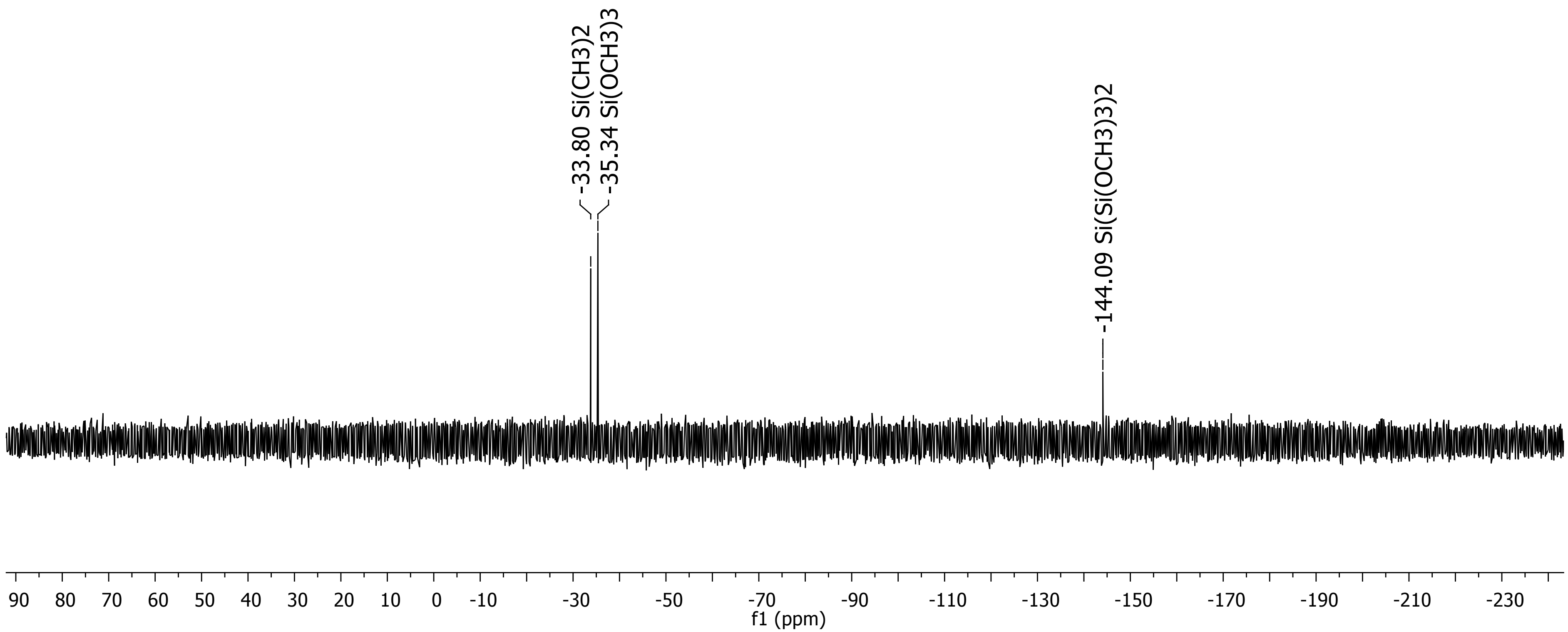

Figure S8. ^{29}Si-INEPT NMR spectrum of 2,2,3,3,5,5,6,6-octamethyl-1,1,4,4-tetrakis(trimethoxysilyl)cyclohexasilane (**5**) (C_6D_6 solution, 40 MHz, RT, ppm)

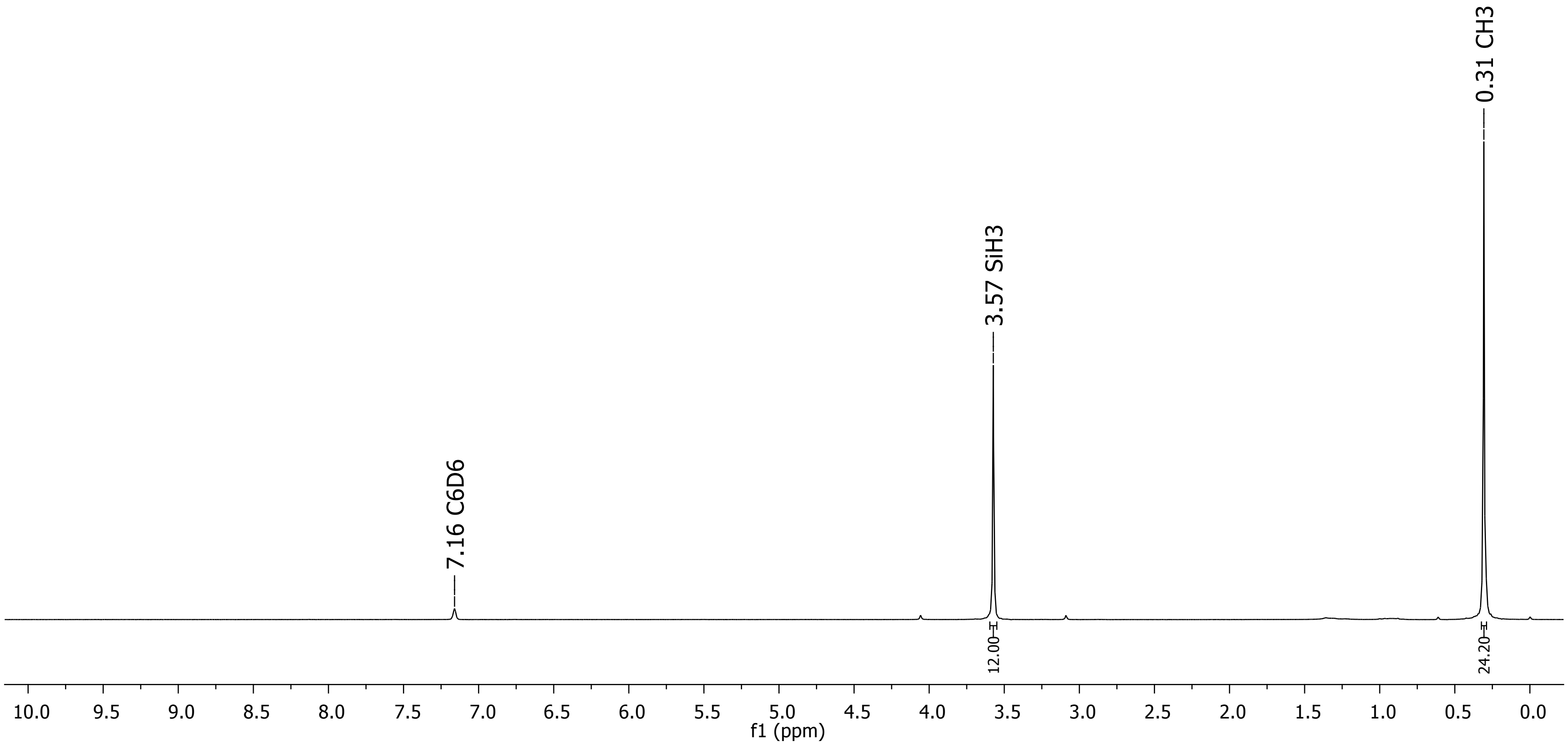

Figure S9. ^{1}H NMR spectrum of 2,2,3,3,5,5,6,6-octamethyl-1,1,4,4-tetrasilylcyclohexasilane (**5**) (C_6D_6 solution, 200 MHz, RT, ppm)

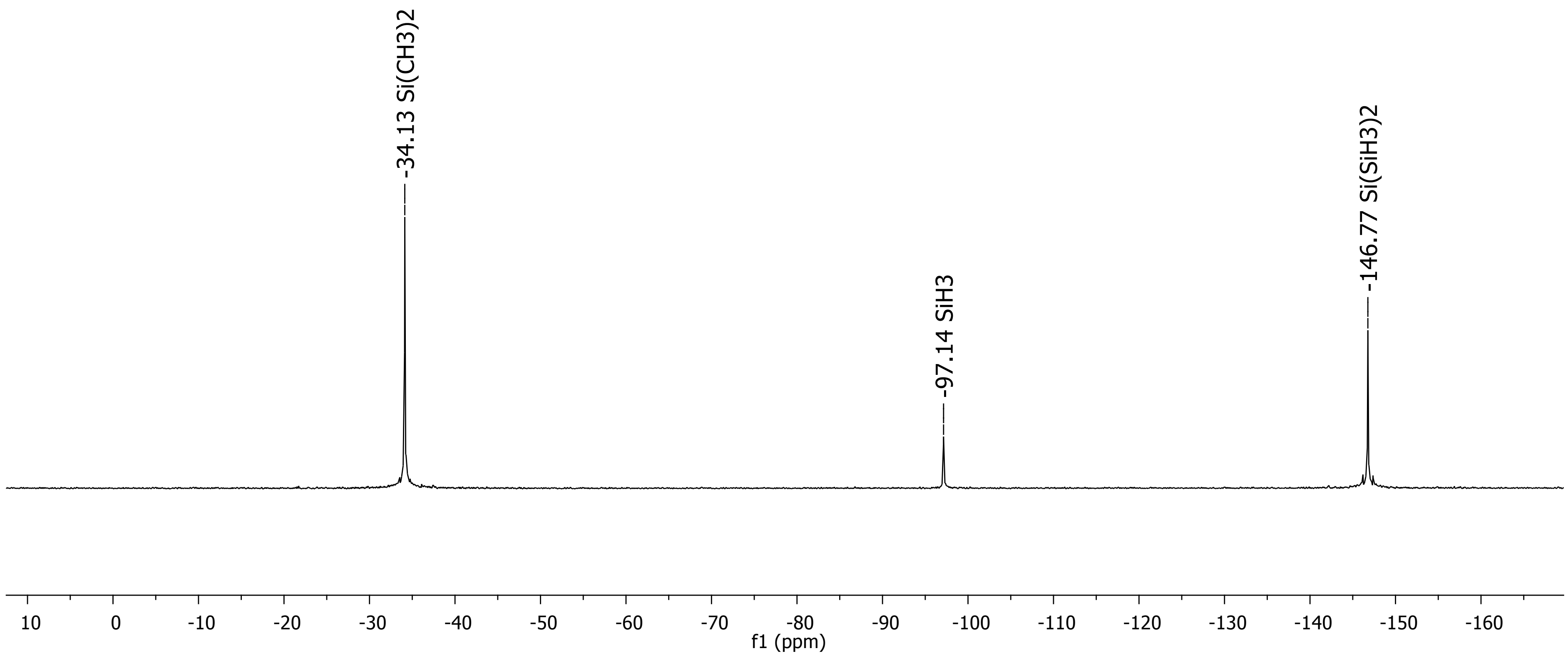

Figure S10. ^{29}Si-INEPT NMR spectrum of 2,2,3,3,5,5,6,6-octamethyl-1,1,4,4-tetrasilylcyclohexasilane (**5**) (C_6D_6 solution, 40 MHz, RT, ppm)

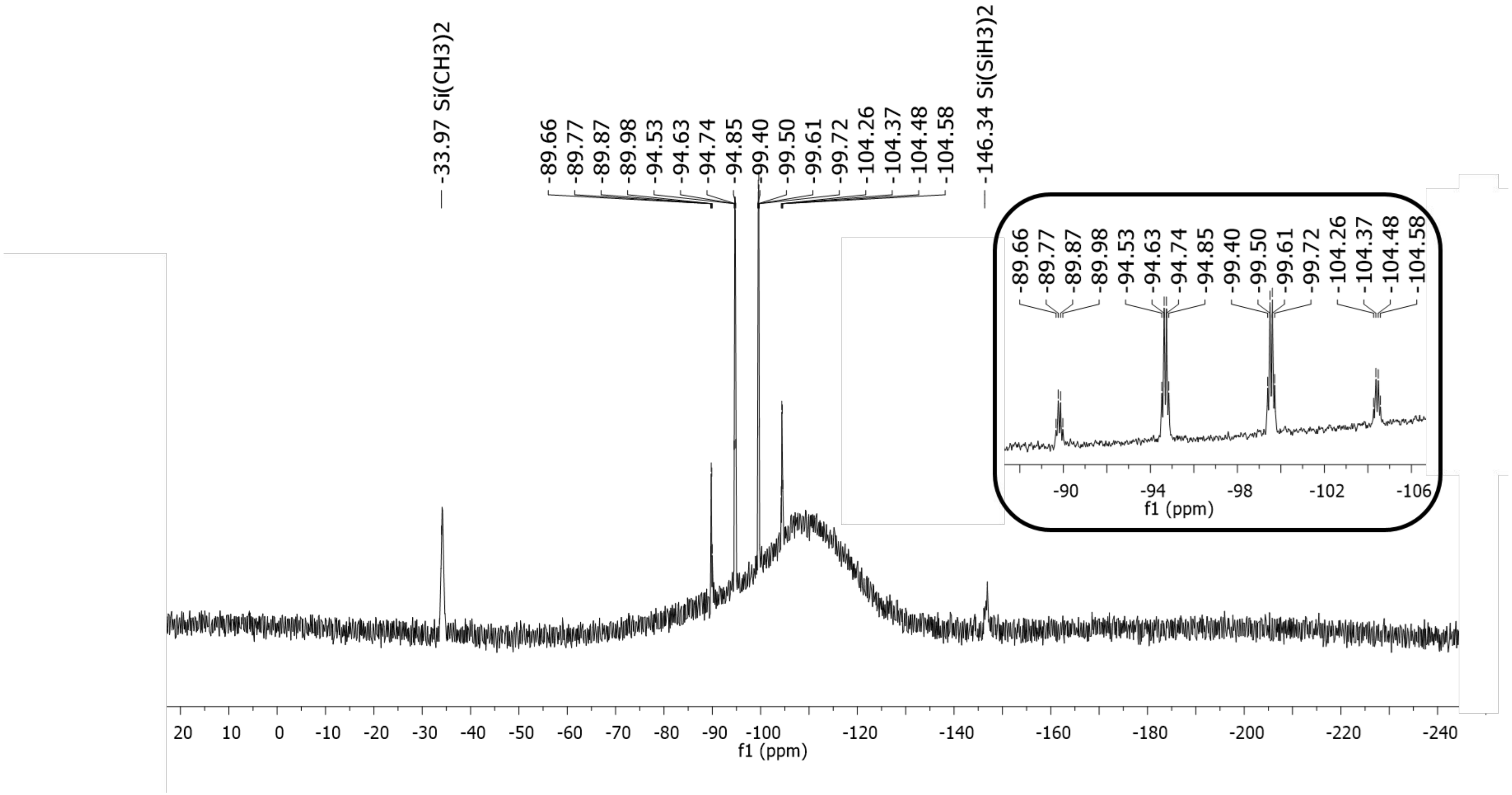

Figure S11. Proton-coupled ^{29}Si NMR spectrum of 2,2,3,3,5,5,6,6-octamethyl-1,1,4,4-tetrasilylcyclohexasilane (**5**) (C_6D_6 solution, 40 MHz, RT, ppm)

Oligomerized Molecules

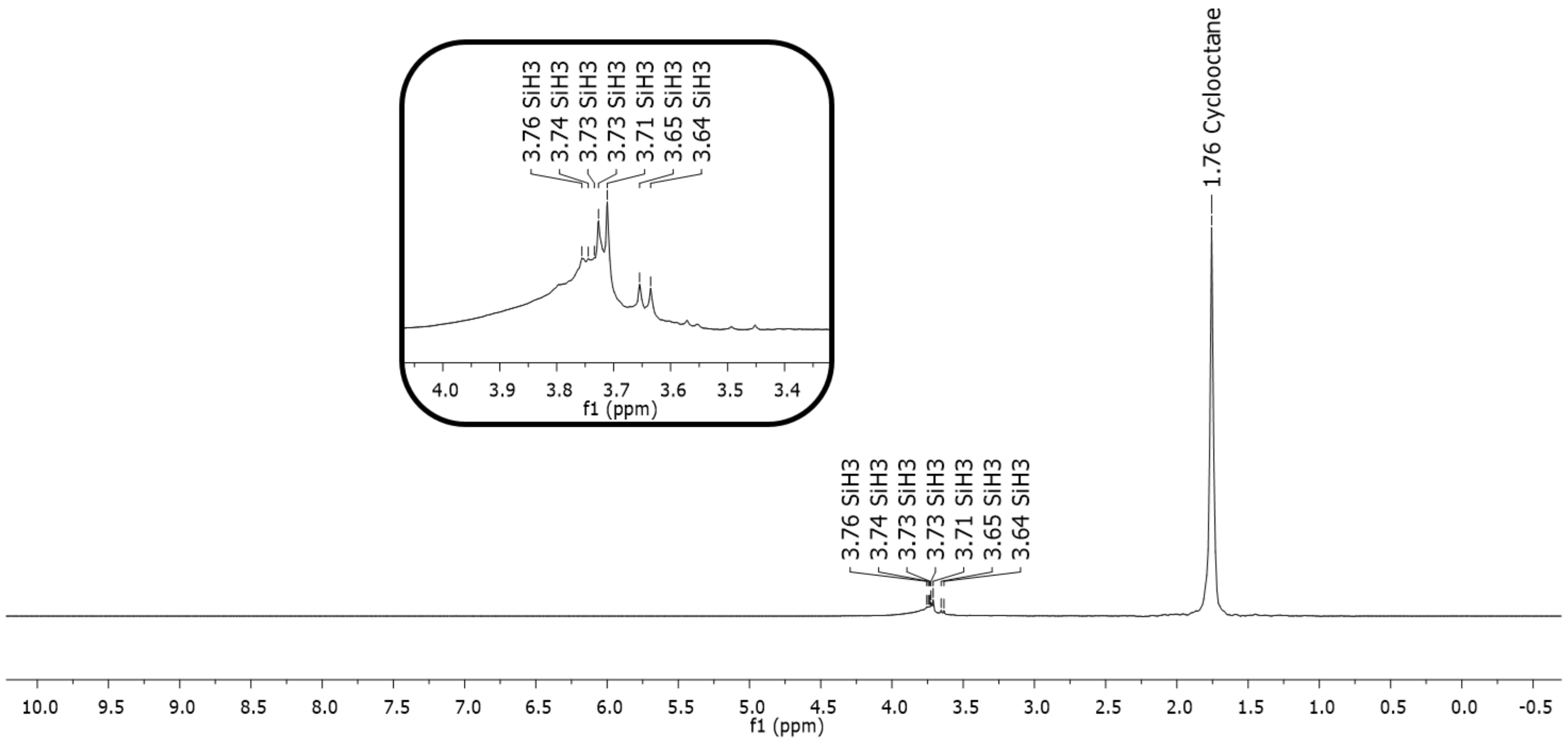

Figure S12. ^{1}H NMR spectrum of oligomerized **2,2-**disilyltrisilane (**NPO**) (D_2O capillary, cyclooctane solution, RT, ppm, 200 MHz)

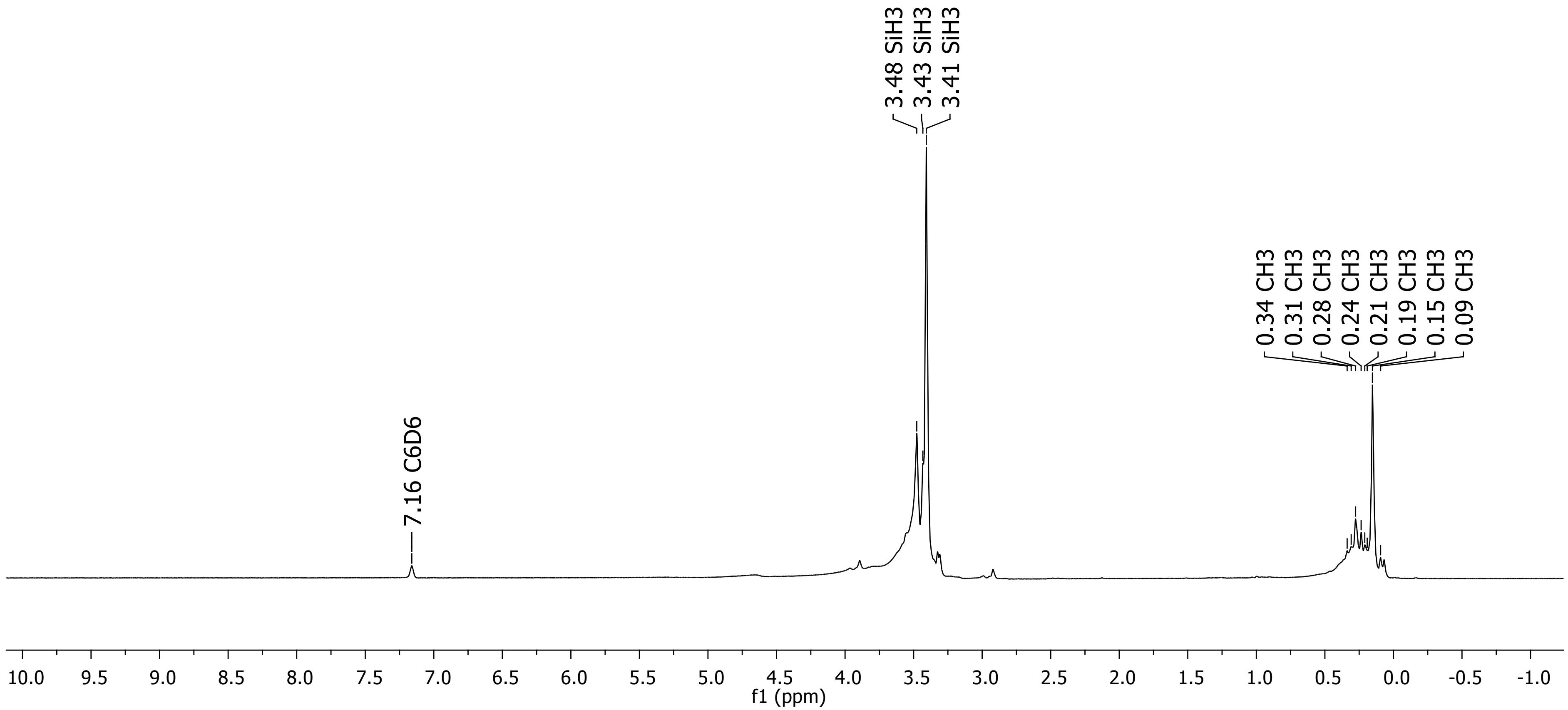

Figure S13. ^{1}H NMR spectrum of oligomerized 2-methyl-2-silyltrisilane (**6**) (C_6D_6 solution, RT, ppm, 200 MHz)

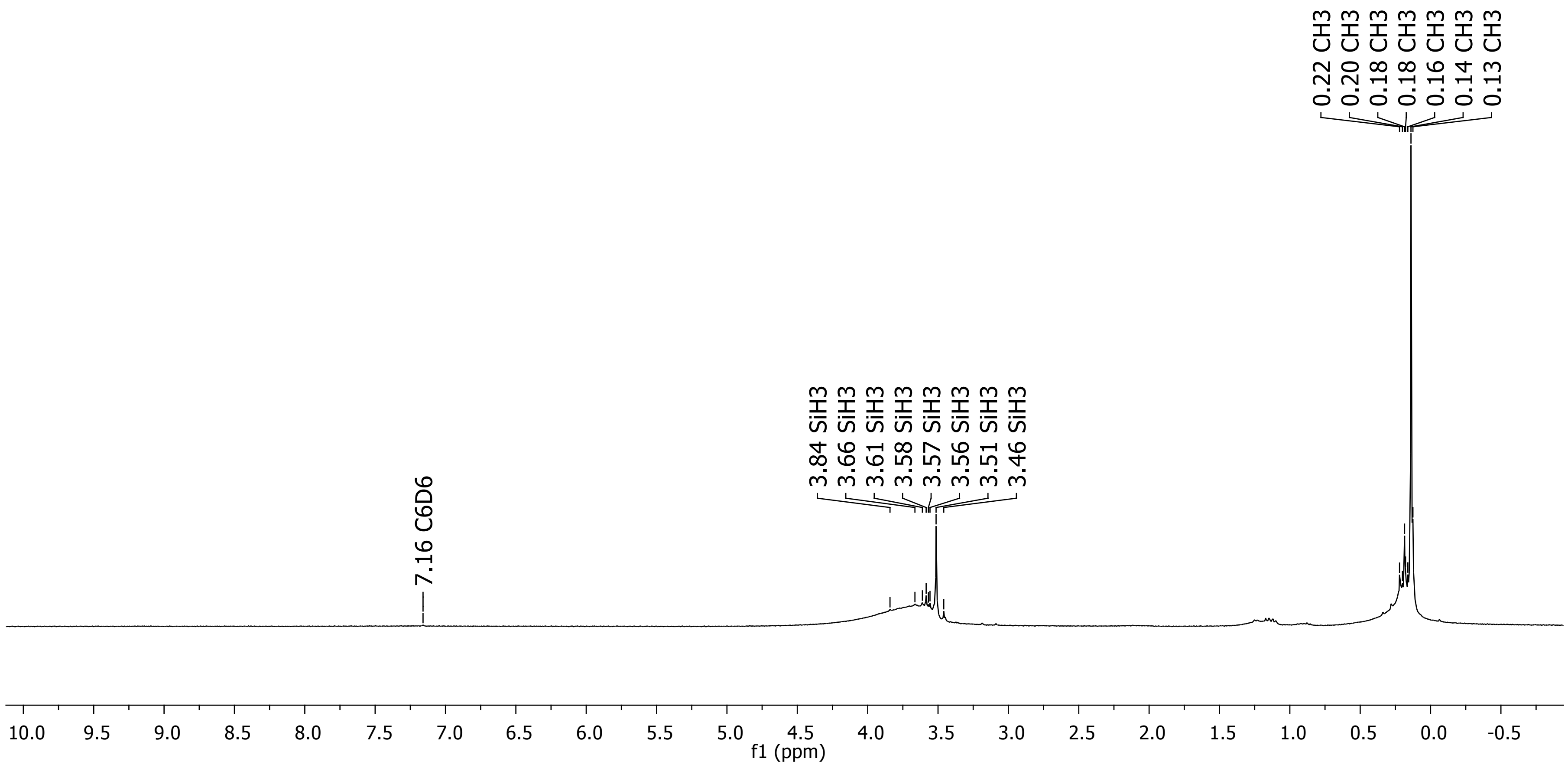

Figure S14. ^{1}H NMR spectrum of oligomerized 1,1,1-trimethyl-2,2-disilyltrisilane (**7**) (C_6D_6 solution, RT, ppm, 200 MHz)

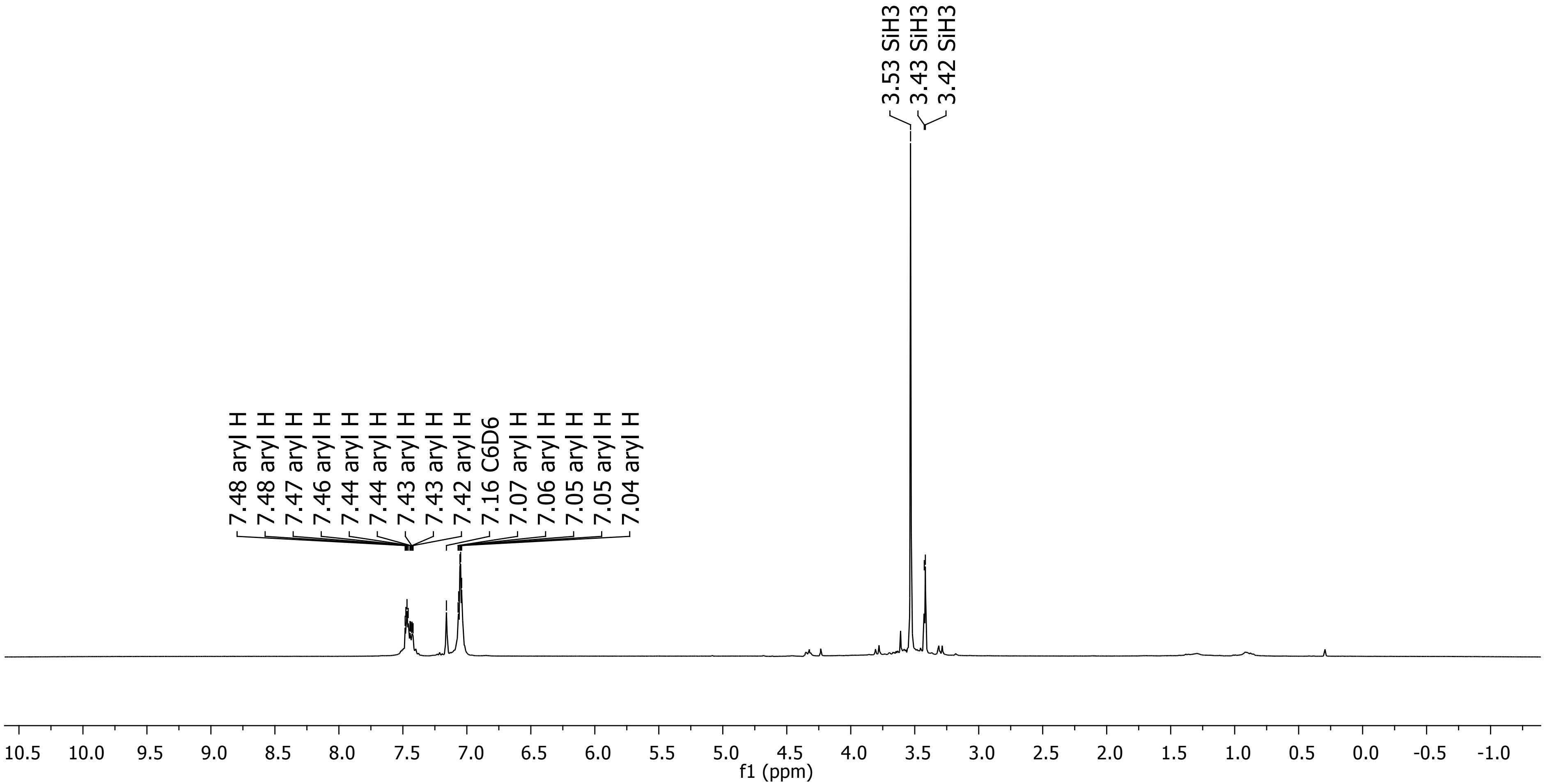

Figure S15. ^{1}H NMR spectrum of oligomerized 2-phenyl-2-silyltrisilane (**8**) (C_6D_6 solution, RT, ppm, 200 MHz)

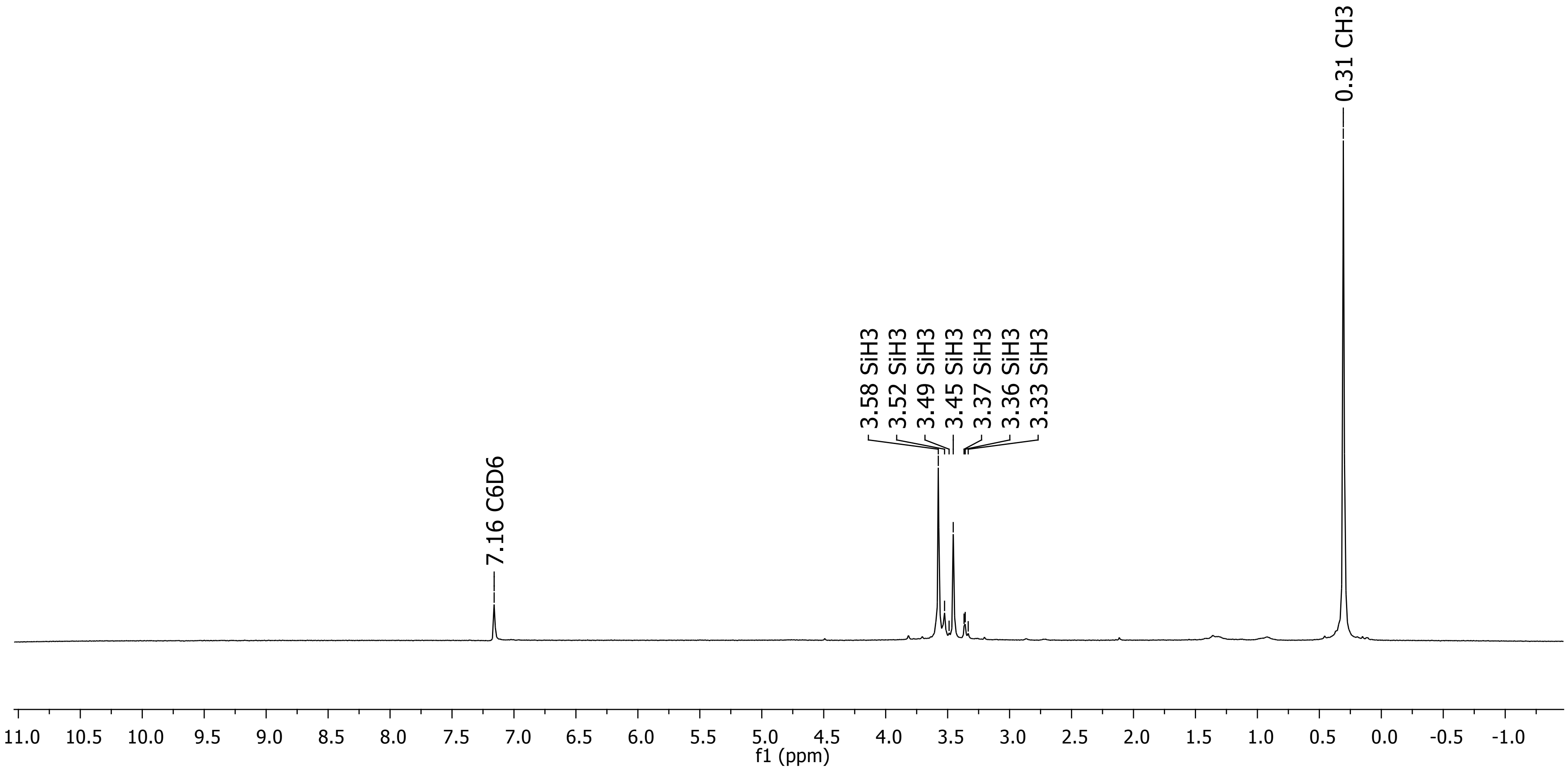

FigureS16. ^{1}H NMR spectrum of oligomerized 2,2,3,3,5,5,6,6-octamethyl-1,1,4,4-tetrasilylcyclohexasilane (**9**) (C_6D_6 solution, RT, ppm, 200 MHz)

UV/Vis spectrometry – Precursor Molecules

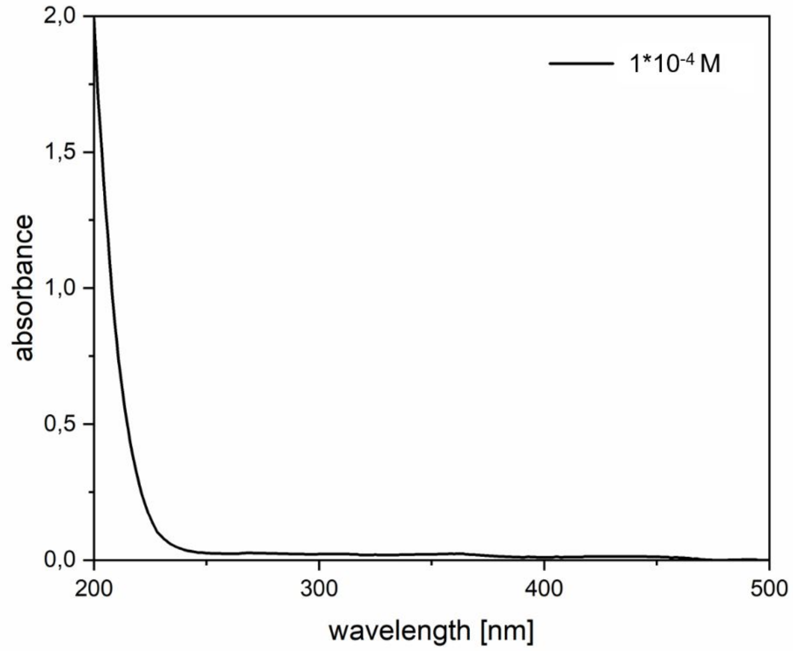

Figure S17. UV/Vis spectra of **1** (c = 1*10^{-4} M, solvent = *n*-hexane)

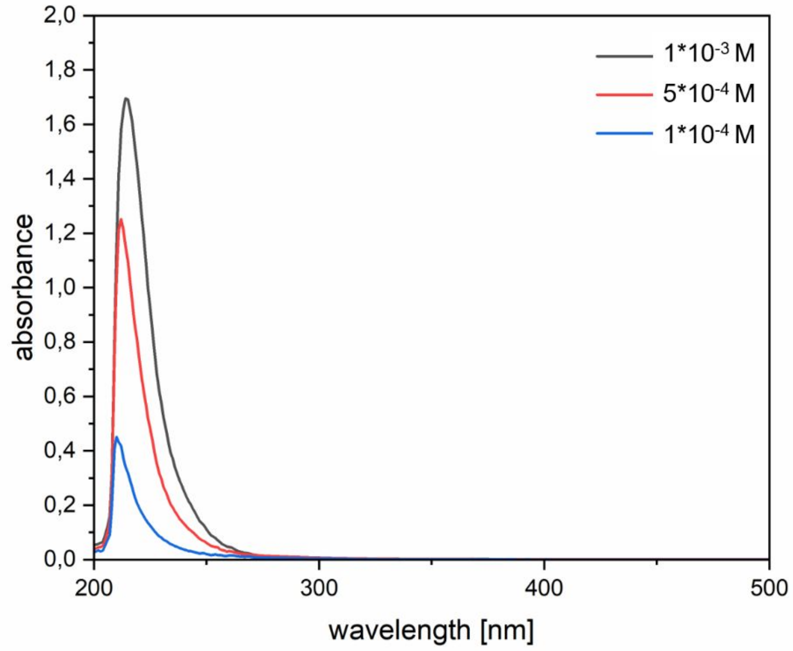

Figure S18. UV/Vis spectra of **2** (black: c = 1*10^{-3} M; red: c = 5*10^{-4} M; blue: c = 1*10^{-4} M; solvent = *n*-hexane)

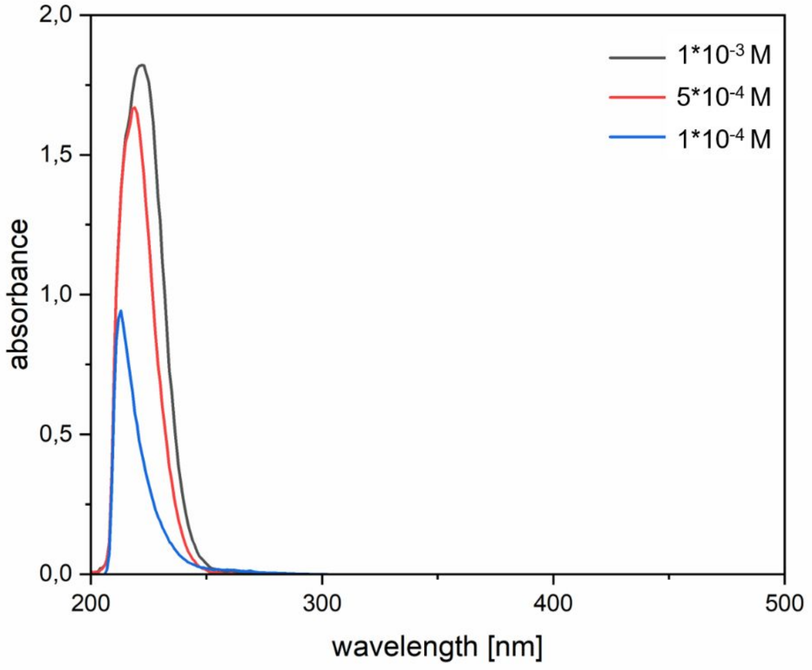

Figure S19. UV/Vis spectra of **3** (black: c = 1*10^{-3} M; red: c = 5*10^{-4} M; blue: c = 1*10^{-4} M; solvent = *n*-hexane)

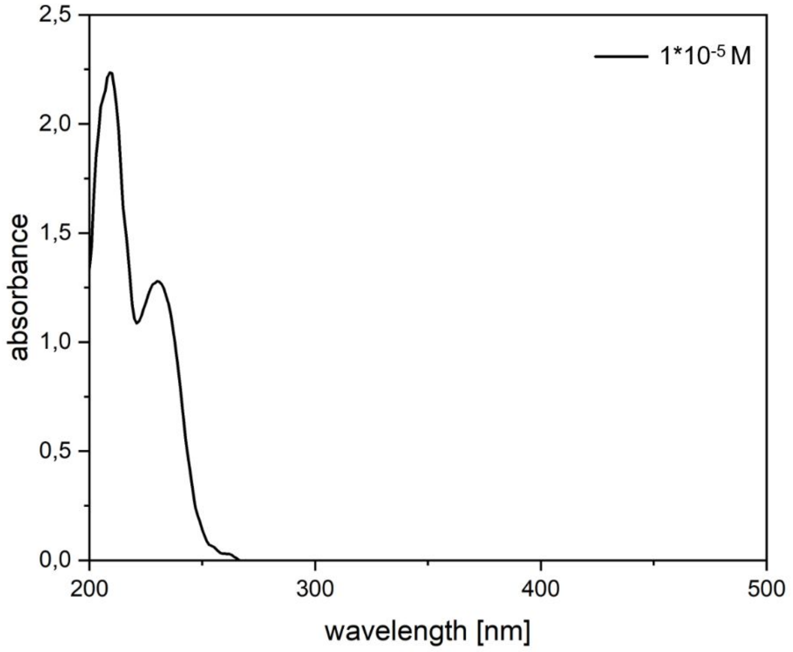

Figure S20. UV/Vis spectra of **4** (c = 1*10^{-5} M; solvent = *n*-hexane)

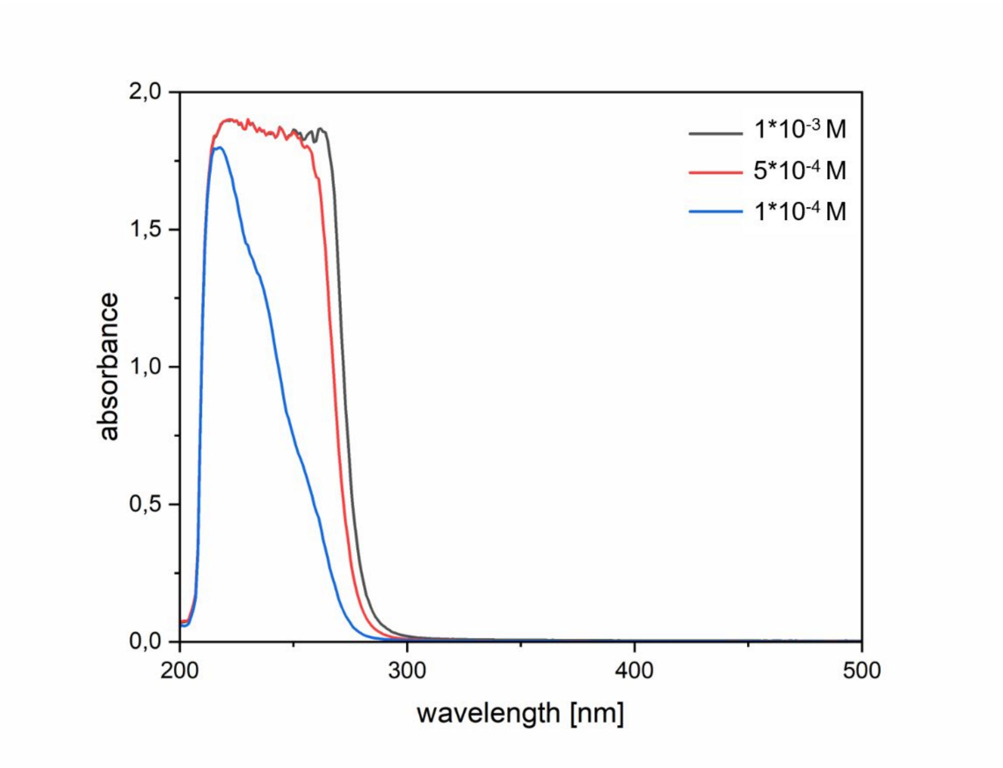

Figure S21. UV/Vis spectra of **5** (black: c = 1*10^{-3} M; red: c = 5*10^{-4} M; blue: c = 1*10^{-4} M; solvent = *n*-hexane)

Crystallographic Data

Table S1. Crystallographic data of compound **5**.

**Compound**	**5** (CCDC: 2262255)
Empirical formula	$C_8H_{36}Si_{10}$
Formula weight [g/mol]	413.27
Temperature/K	100.03
Crystal system	monoclinic
Space group	$P2_1/n$
a/Å	8.1493(6)
b/Å	14.7720(11)
c/Å	10.7914(7)
α/°	90
β/°	92.687(2)
γ/°	90
Volume/Å^3	1297.66(16)
Z	2
ρ_{calc}g/cm^3	1.058
μ/mm^{-1}	0.495
F(000)	448.0
Crystal size/mm^3	0.45 × 0.43 × 0.37
Radiation	MoKα (λ = 0.71073)
2Θ range for data collection/°	5.516 to 65.288
Index ranges	$-12 \leq h \leq 12$, $-22 \leq k \leq 22$, $-15 \leq l \leq 16$
Reflections collected	70242
Independent reflections	4716 [R_{int} = 0.0344, R_{sigma} = 0.0133]
Data/restraints/parameters	4716/0/110
Goodness-of-fit on F^2	1.093
Final R indexes [I>=2σ (I)]	R_1 = 0.0183, wR_2 = 0.0475
Final R indexes [all data]	R_1 = 0.0202, wR_2 = 0.0485
Largest diff. peak/hole / e Å^{-3}	0.36/-0.23

Thin Layer Materials

Deposition and Solution Parameters

Thin layer materials were spin coated on glass substrates (25 x 25 mm). Prior to the spin coating, the substrates were cleaned with deionized water, acetone and sonication in isopropanol (2 h). For all compounds different solutions were made. The optimized conditions can be seen in Table 1. The spin coating speed for depositing the layers was varied from 3000 rpm to 9000 rpm at 10-20 s. Further spin coating parameters and their optimization are depicted in Table 2. The successfully spin coated materials were then deposited at 500 °C to achieve amorphous Si/Si-C layers.

Table S2. Optimized conditions for the prepared spin coating solutions. (CO = cyclooctane).

Compound	Concentration [wt%]	Solvent	Volume [mL]	Temperature [°C]
**NPO**	50	toluene	0.20	RT
**6**	50	toluene	0.20	-30
**7**	50	toluene + 10 wt% CO	0.24	-30
**8**		toluene + 10 wt% CO	0.24	-30
**9**	15	THF	RT	RT

Table S3. Optimized spin coating parameters of the different solutions.

Compound	Volume [mL]	Speed [rpm]	Time [s]	Depositing temperature [°C]
**NPO**	0.20	9000	20	500
**6**	0.20	9000	20	500
**7**	0.24	3000	10	500
**8**	0.24	4000	10	500
**9**	0.24	4000	10	500

Optical Properties

UV/Vis-spectroscopy was recorded with an UV/Vis-spectrometer Lambda 35 from Perkin Elmer. The settings can be seen in Table 3. The absorption spectra were measured in thin films on glass substrates after spin coating and deposition at 500 °C.

Table S4. Settings for absorption measurements of thin layer materials.

**Start wavelength [nm]**	800
**End wavelength [nm]**	300
**Slit witdh [nm]**	1.0
**Scan speed [nm/min]**	240
**Data interval [nm]**	1.0

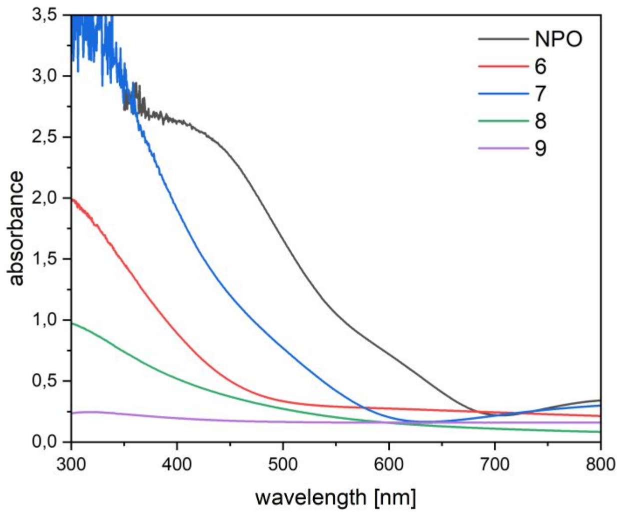

Figure S22. UV/Vis spectra of thin layer materials of **NPO** and **6** – **9**.

Images of the layer surfaces with a magnification of 200x were obtained by light microscopy. Therefore, a Light Microscope BX60 from Olymp with an attached camera (Olymp). Following images (Figure 20-24) visualize the surface of each layer material in different concentrations/solvents.

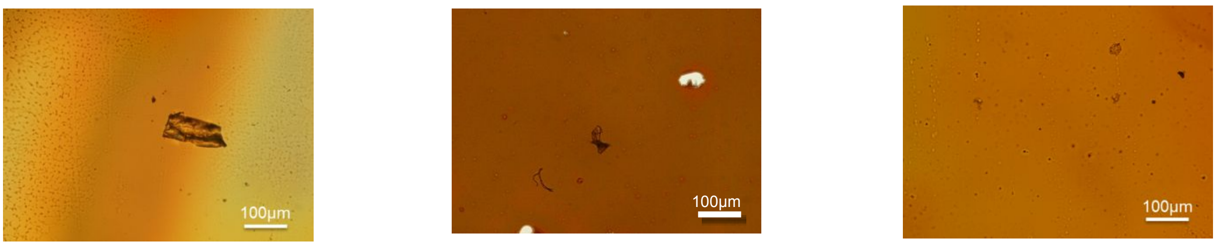

Figure S23. Microscopic images with 200x magnification of **NPO** in different concentrations and solvents (left: 30 wt% in toluene; middle: 50 wt% in toluene + 10 wt% CO; right: 50 wt% in toluene).

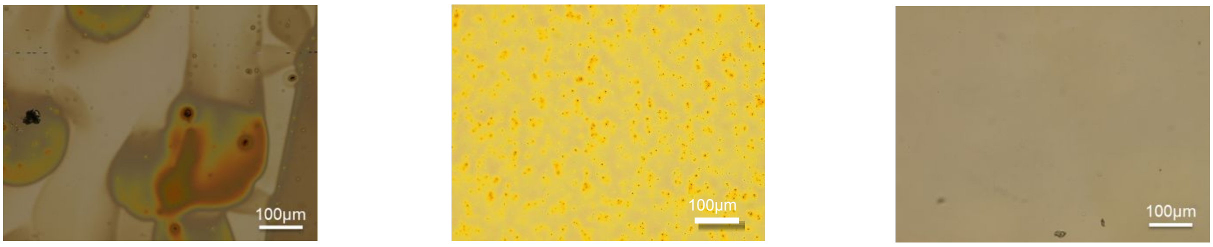

Figure S24. Microscopic images with 200x magnification of **6** in different concentrations and solvents (left: 70 wt% in CO; middle: 50 wt% in toluene; right: 50 wt% in toluene filtrated).

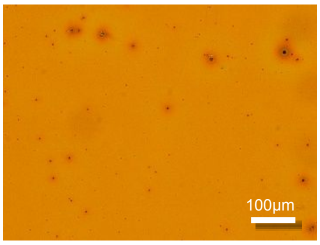

Figure S25. Microscopic image with 200x magnification of **7** (50 wt% in toluene + 10 wt% CO).

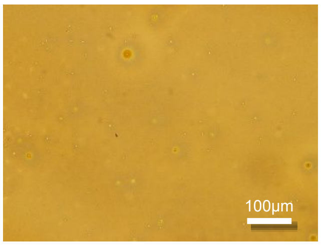

Figure S26. Microscopic image with 200x magnification of **8** (50 wt% in toluene + 10 wt% CO).

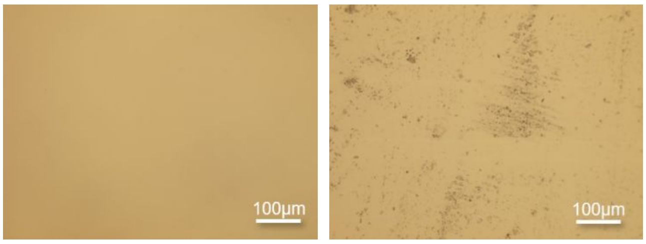

Figure S27. Microscopic image with 200x magnification of the same deposited layer of 15 wt% **9** (left: homogenous; right inhomogenous parts of the thin layer material)

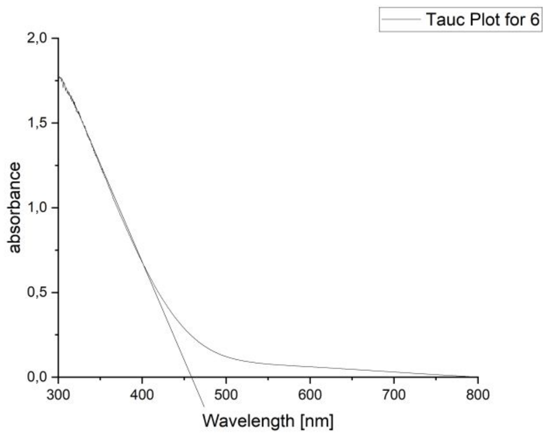

Figure S28. Tauc plot for determining the band gap of a thin film for **6**.

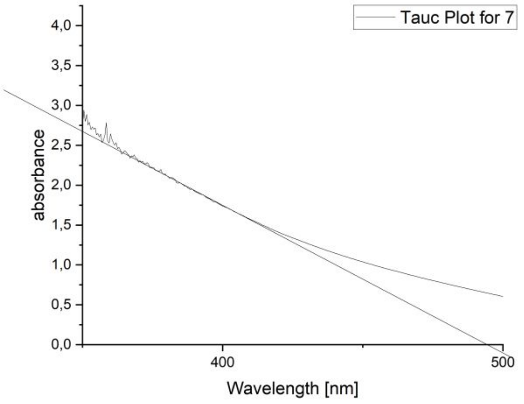

Figure S29. Tauc plot for determining the band gap of a thin film for **7**.

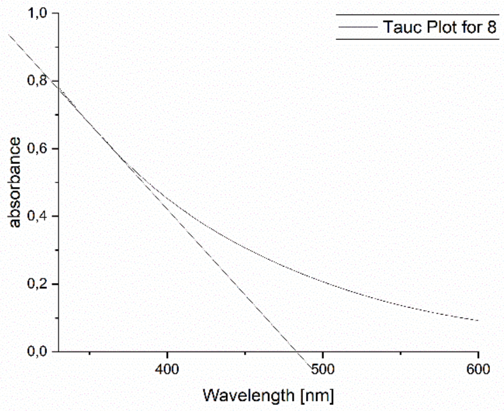

Figure S30. Tauc plot for determining the band gap of a thin film for **8**.

Layer Thickness and Elemental Composition

Spectroscopic Ellipsometry:

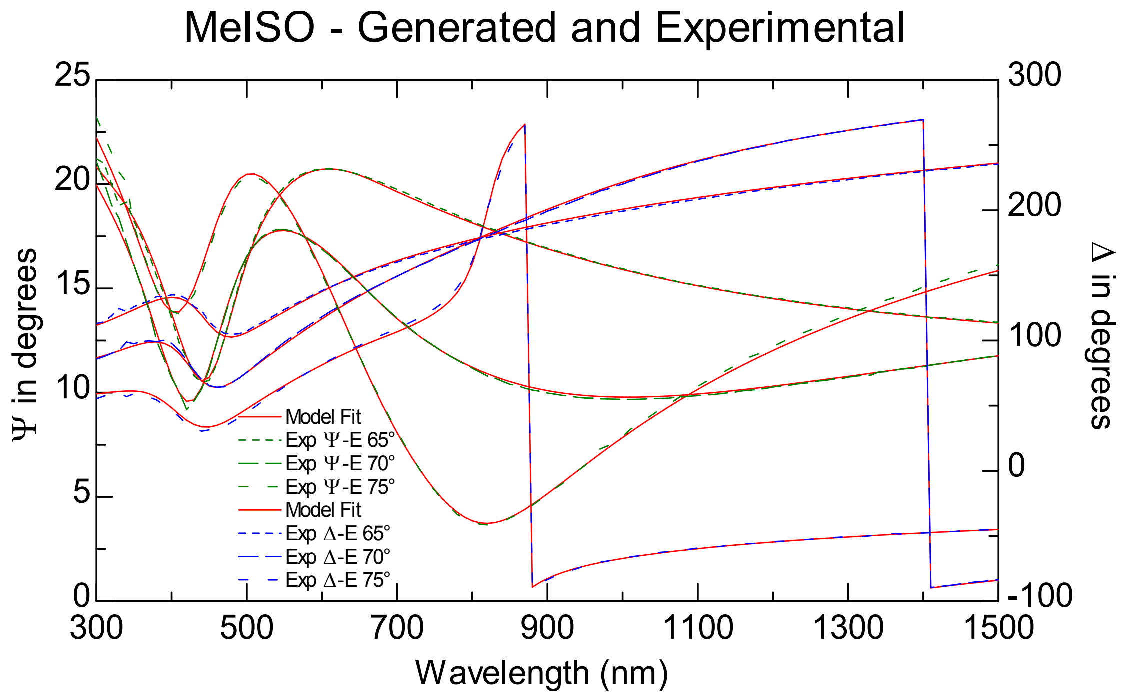

Figure S31. Ellipsometric parameter Ψ and Δ of thin layer **MeISO**. The dotted green and blue lines show the measured values, the red curves show the fitted calculated data. The model used for the calculation consisted of a pole site in the UV outside the measured spectral range, a DC offset, an (asymmetric) Cody-Lorentz- as well as a much smaller (symmetric) Gaussian broadened-oscillator in the UV-VIS-NIR range and a Drude term for the long-wavelength absorption in the IR.

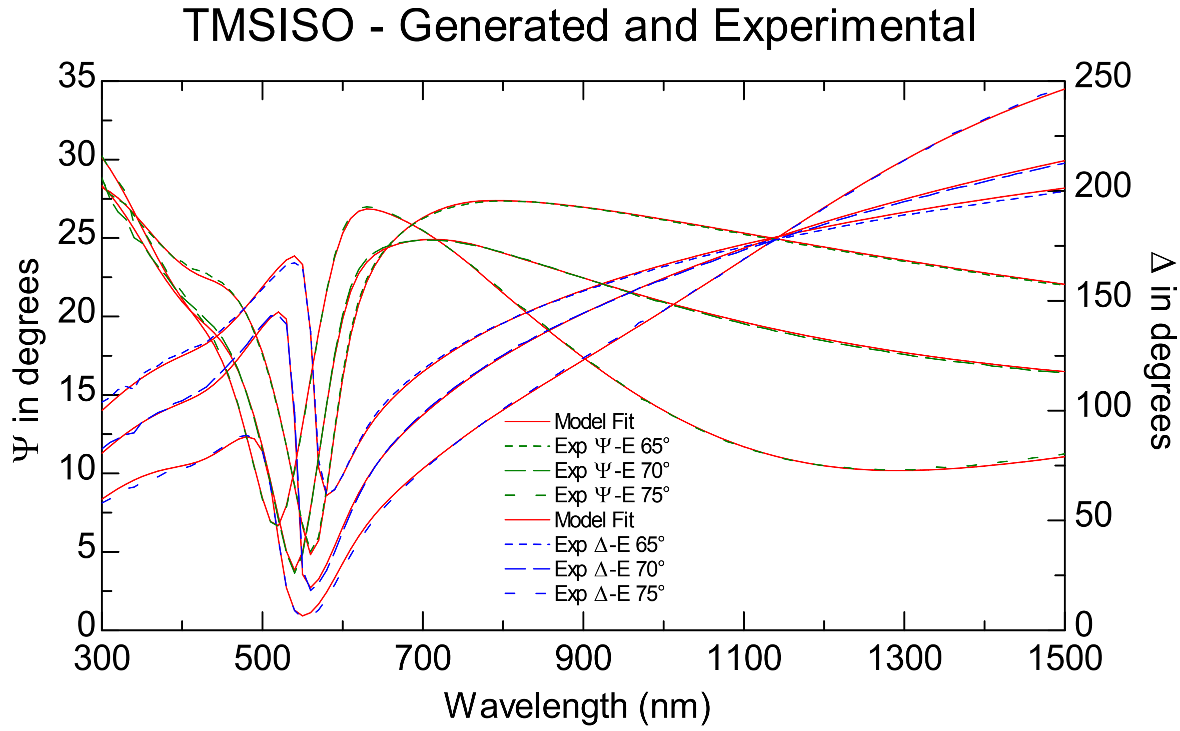

Figure S32. Ellipsometric parameter Ψ and Δ of thin layer **TMSISO**. The dotted green and blue lines show the measured values, the red curves show the fitted calculated data. The model used for the calculation consisted of a pole site in the UV outside the measured spectral range, a DC offset, an (asymmetric) Cody-Lorentz- as well as a much smaller (symmetric) Gaussian broadened-oscillator in the UV-VIS-NIR range and a Drude term for the long-wavelength absorption in the IR.

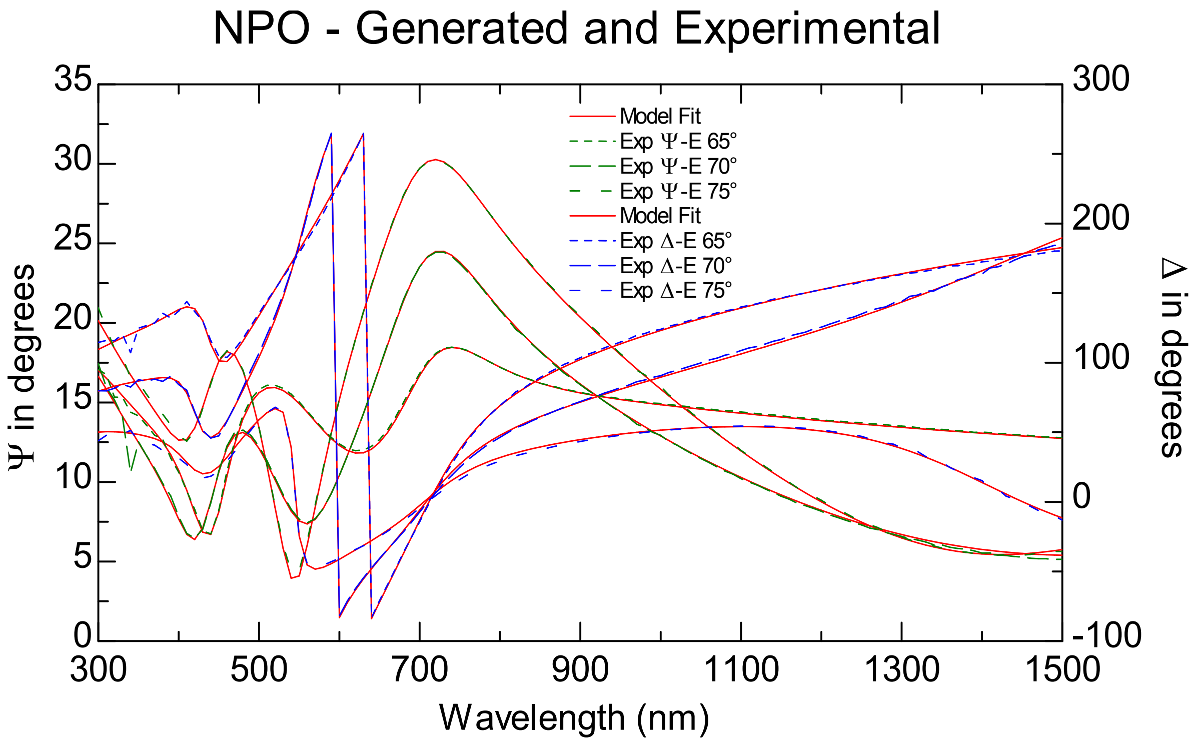

Figure S33. Ellipsometric parameter Ψ and Δ of thin layer **NPO**. The dotted green and blue lines show the measured values, the red curves show the fitted calculated data. The model used for the calculation consisted of a pole site in the UV outside the measured spectral range, a DC offset, an (asymmetric) Cody-Lorentz- as well as a much smaller (symmetric) Gaussian broadened-oscillator in the UV-VIS-NIR range. Here, no Drude term was necessary to describe the IR part of the absorption.

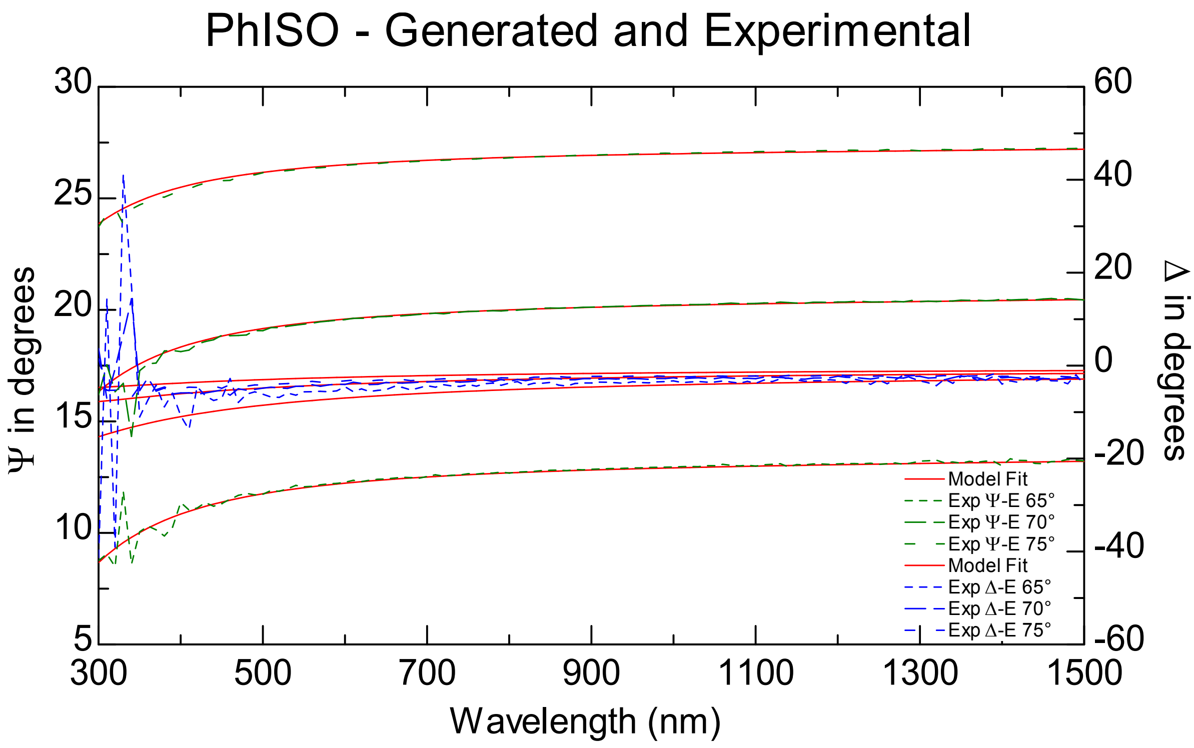

Figure S34. Ellipsometric parameter Ψ and Δ of thin layer **PhISO**. The dotted green and blue lines show the measured values, the red curves show the fitted calculated data. The model used for the calculation consisted of a pole site in the UV outside the measured spectral range and a DC offset. No absorption could be found for this layer. It is to say that the very small thickness of this layer additionally to general restrictions of ellipsometric sensitivity to small absorption sets a strong limit for absorption determination.

Elemental analysis and further images of the surface layer were obtained by Freskida Goni via SEM/EDX. SEM micrographs and EDX spectra were collected using Tescan VEGA 3 SEM (Oxford Instruments plc, Abingdon, United Kingdom) with tungsten source filament working at 20 kV. For SEM imagines, a resolution of 5 µm and a working distance of around 15 mm was used, and the EDX spectra were collect4ed at a 0-10 keV scale. Prior the analysis, the samples were sputter-coated with gold. Table 5 summarizes the obtained data in aspect of elemental composition of the different layers. Small amounts of different elements were omitted. The amount of oxygen was omitted as well, to be able to compare the silicon and carbon amount respectively

Table S5. Summary of the silicon, carbon and oxygen amount of different thin layer.

	O[%]	C [%]	Si [%]
**NPO**	75.97	0.00	24.03
	75.96	0.00	24.04
	75.82	0.00	24.18
**6**	56.63	4.30	39.06
	56.29	4.05	39.67
	56.33	4.00	39.67
**7**	55.24	2.73	42,03
	54.92	2.56	42.53
	54.73	2.38	42.89
**8**	66.05	6.49	27.46
	63.82	7.62	28.57
	65.30	7.48	27.22

Additionally, images of the surface area of the different layer materials were obtained. Figure 27-31 show the surfaces of the different thin layers.

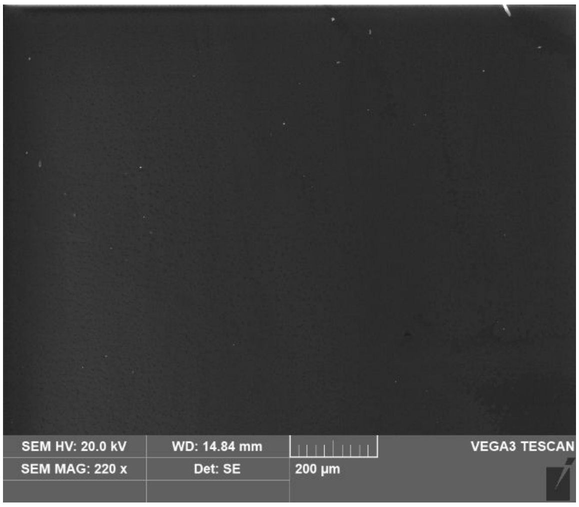

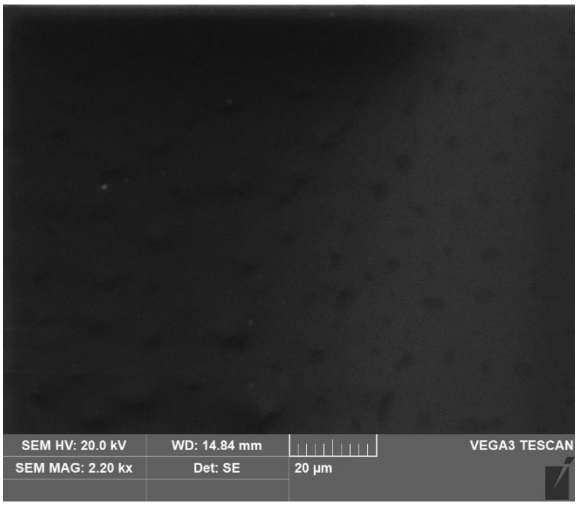

Figure S35. SEM images of thin layers of **NPO** in different magnifications (left: 220x; right: 2.20 kx)

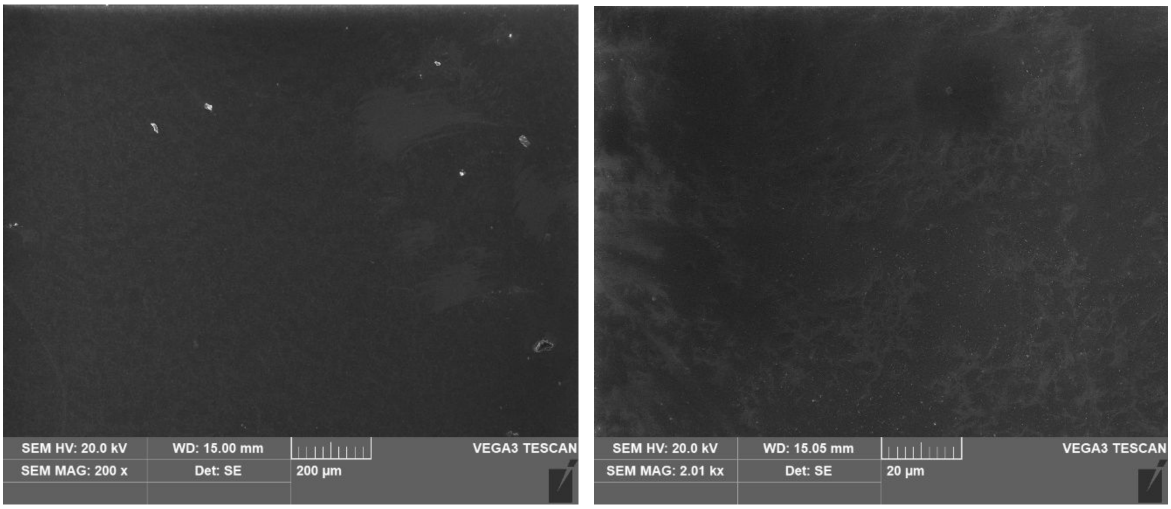

Figure S36. SEM images of thin layers of compound **6** in different magnifications (left: 200x; right: 2.01 kx)

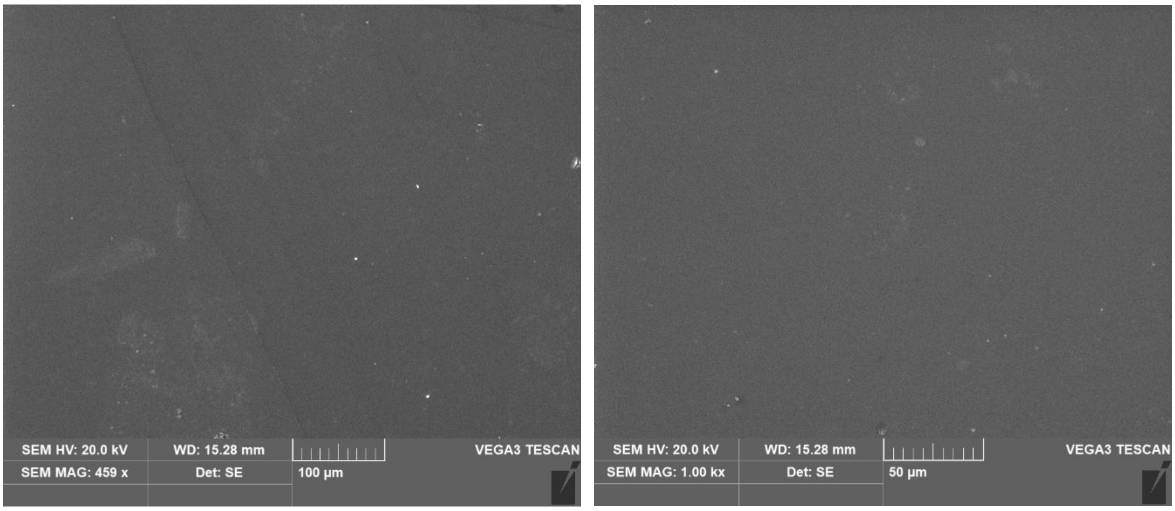

Figure S37. SEM images of thin layers of compound **7** in different magnifications (left: 459x; right: 1.00 kx)

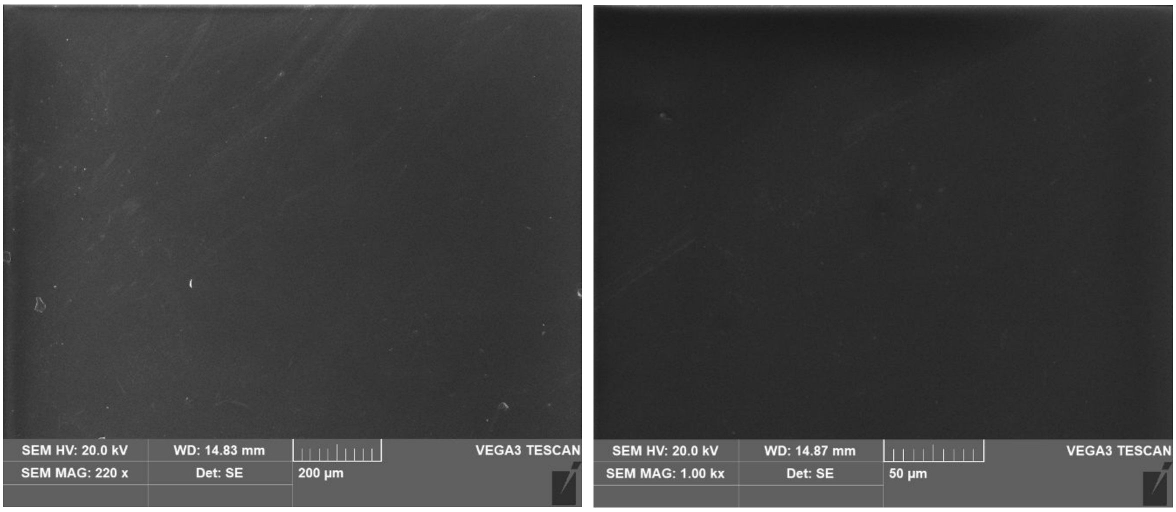

Figure S38. SEM images of thin layers of compound **8** in different magnifications (left: 220x; right: 1.00 kx)

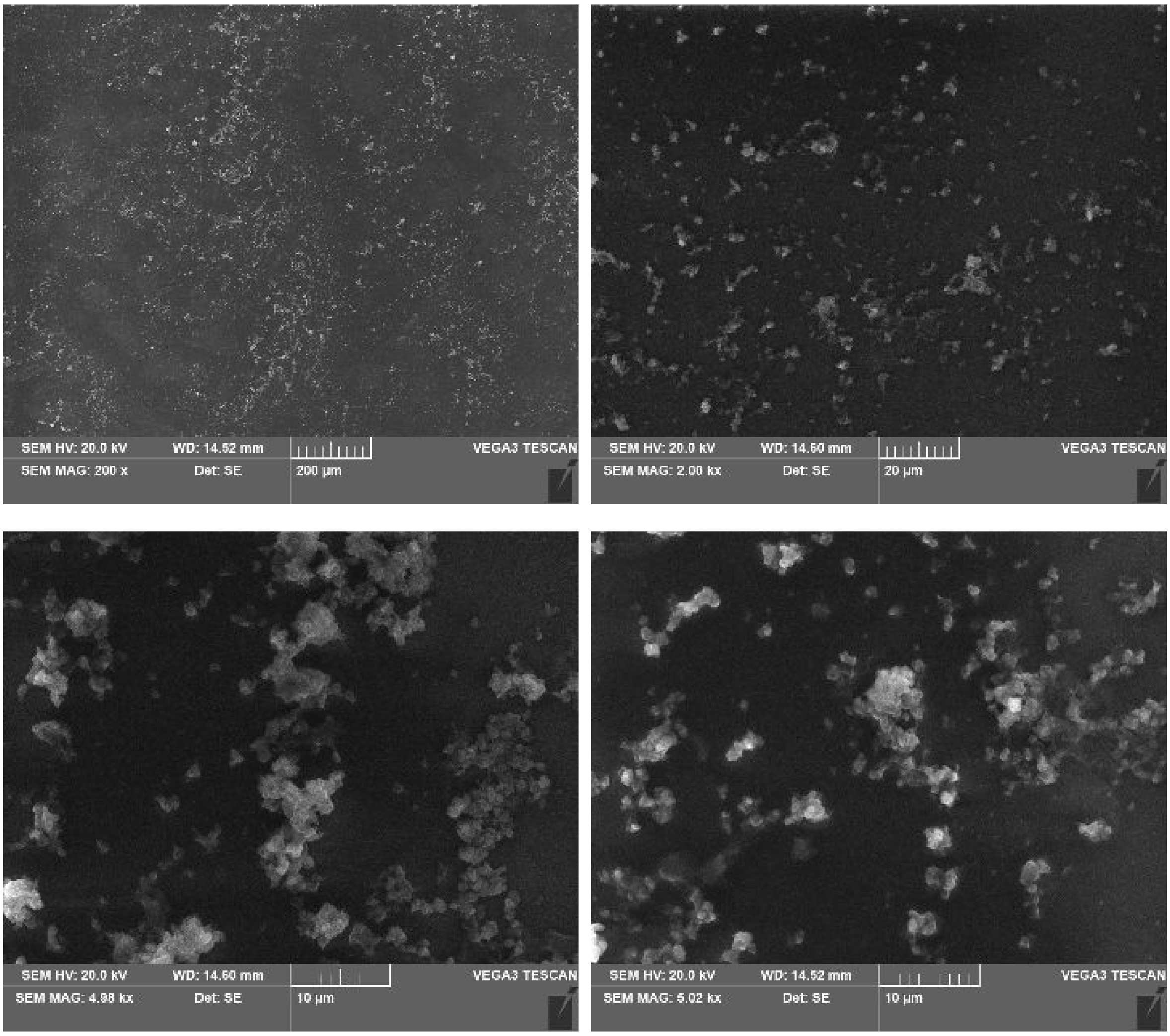

Figure S39. SEM images of thin layers of compound **9** in different magnifications (top left: 200 x; top right: 2.00 kx; bottom left: 4.98 kx; bottom right: 5.02 kx)

Table S6. Surface Composition of the thin layers determined by XPS measurements

	surface composition [at%]						
**sample**	**O**	**C**	**Si**	**Na**	**F**	**N**	**Al**
**MeSI**	30.2 ± 4.3	42.0 ± 8.3	25.2 ± 5.0	1.9 ± 0.8	0.7 ± 0.1	-	-
**NPO**	43.1 ± 1.4	19.5 ± 2.4	37.4 ± 0.9	-	-	-	-
**PHIs**	25.5 ± 6.4	57.8 ± 10.5	13.5 ± 5.6	2.2 ± 0.2	1.2*	0.8*	-
**TMSIS**	34.6 ± 0.7	32.7 ± 8.6	17.2 ± 0.3	0.8 ± 0.3	8.1 ± 9.8	0.8*	6.1 ± 0.0

*...single value, detected on only one measurement spots on the sample

The high carbon values are assigned to carbonaceous compounds such as CO_2 etc., which were adsorbed to the surfaces during storage under atmospheric conditions. The adsorption of small molecules from the atmosphere is a well-known phenomenon in surface analysis. The origin of the carbon signal becomes obvious by considering the NPO sample, on which 19.5 at% C were detected although the thin layer investigated consisted of a neopentasilane oligomer.

Table S7. Elemental Analysis of the oligomers performed after photolysis.

**oligomer 6**	**%N**	**%C**	**%H**	**%S**
1.		9.25	8.96	
2.		9.55	9.25	
3.		9.86	9.55	
**MW**	**n.n.**	**9.55**	**9.25**	**n.n.**
*C/H value of the precursor **2***		*8.80*	*8.86*	
**oligomer 7**	**%N**	**%C**	**%H**	**%S**
1.		8.52	10.23	
2.		9.56	9.89	
3.		8.95	9.78	
**MW**	**n.n.**	**9.01**	**9.97**	**n.n.**
C/H value of the precursor 3		*18.52*	*9.32*	
**oligomer 8**	**%N**	**%C**	**%H**	**%S**
1.		18.95	7.56	
2.		19.06	7.05	
3.		18.86	7.68	
**MW**	**n.n.**	**18.96**	**7.43**	**n.n.**
C/H value of the precursor 4		*36.30*	*7.11*	
**oligomer 9**	**%N**	**%C**	**%H**	**%S**
1.		20.36	6.10	
2.		20.69	5.48	
3.		20.26	6.78	
**MW**	**n.n.**	**20.44**	**6.12**	**n.n.**
C/H value of the precursor 5		*23.25*	*8.78*	

DFT Calculations

Computational Methods

The optimization of conformations for **1-5** was performed with the composite DFT method PBEh-3c.[1] In this method, the electronic PBE0 energy is corrected by Grimme's D3BJ dispersion correction, and the geometrical counterpoise energy (gCP) is added for correction of the basis set superposition error. The basis set def2-SVP is applied in this method. The geometries were checked to be minima at the potential energy surface by calculation of the harmonic frequencies. The calculations were performed in the solvent *n*-hexane using the conductor-like polarizable continuum model (CPCM) for solvation.[2] Time-dependent DFT (TD-DFT) was applied for the computation of 15 vertical excitations, and the UV/Vis spectrum was then simulated from these data by Gaussian broadening of the peaks with a half width at half height of 10 nm using the program gabedit. Molecular orbitals were generated by the program Avogadro,[3] and the UV spectrum was simulated by the program gabedit.[4] The program ORCA 5.0.3 was used for all DFT calculations.[5]

Simulated UV/Vis Spectra and Molecular Orbitals

The simulated UV/Vis spectra of compounds **1-5** (Figure S41) are systematically blue-shifted compared to the experimental spectrum, which is a known behavior of the PBEh-3c functional. Nevertheless, general features of the computed spectra compare well with the experiment, and the character of the bands can be explained by interpreting the molecular orbitals (see Table S8 and Figure S40). In general, the S1 band is very weak with the exception of compounds **2** (R=Me) and **5** (Ring structure). The high-intensity band as well as the S1 bands are interpreted in the following text.

Compounds **1** (R=SiH_3) and **3** (R=$SiMe_3$) provide similar spectra, although the very weak S1 band is red-shifted in compound **3** relative to **1** by ca. 10 nm, which is rationalized by the $SiMe_3$ instead of SiH_3 group. The same effect can be seen when methyl is introduced in compound **2** (R=Me), where the main effect is also a higher intensity of the bands. The ring compound **5** increases this behavior, and the high-intensity of the HOMO-LUMO band also from a σ–σ* transition of the ring system. Additionally, this band has approximately the same wavelength as in compound **2**. Compound **4** (R=Ph) shows a different characteristic, as both S1 and S2 bands consist of linear combination of the frontier orbitals, where the HOMO-LUMO excitation is the second S2 band with high intensity. Because the respective orbitals are localized at the phenyl substituent only, the expected high intensity of a normal π–π* transition occurs in compound **4** at the intense S2 band, which is strongly shifted to the red relative to compound **2**.

Table S8. Most important simulated PBEh-3c UV vertical excitations: wavelength (in nm), oscillator strength (italic, in brackets) and respective orbital contributions with squared LCAO coefficients c^2 (in brackets). The first S1 excitation as well as the most intense band is tabulated. H denotes HOMO, L denotes LUMO.

Compound<br>Band	**1**	**2**	**3**	**4**	**5**
S_1	176.4<br>(*0.000002*)<br>H-2 → L+2 (0.17)<br>H → L (0.15)	182.8<br>(*0.0608*)<br>H → L (0.87)	185.4<br>(*0.0066*)<br>H → L (0.77)<br>52a → 57a (0.14)	223.2<br>(*0.0032*)<br>H-1 → L (0.48)<br>H → L+1 (0.44)	218.4<br>(*0.1401*)<br>H → L (0.93)
$S_{intense}$	S13:<br>161.6<br>(*0.2449*)<br>H → L+5 (0.95)	S5:<br>166.5<br>(*0.3113*)<br>H-1 → L+2 (0.37)<br>H → L+1 (0.32)	S13:<br>165.0<br>(*0.2021*)<br>H → L+5 (0.84)	S2:<br>206.9<br>(*0.3916*)<br>H → L (0.84)	S4:<br>205.8<br>(*0.1378*)<br>H → L+2 (0.55)<br>H-1 → L+1 (0.17)

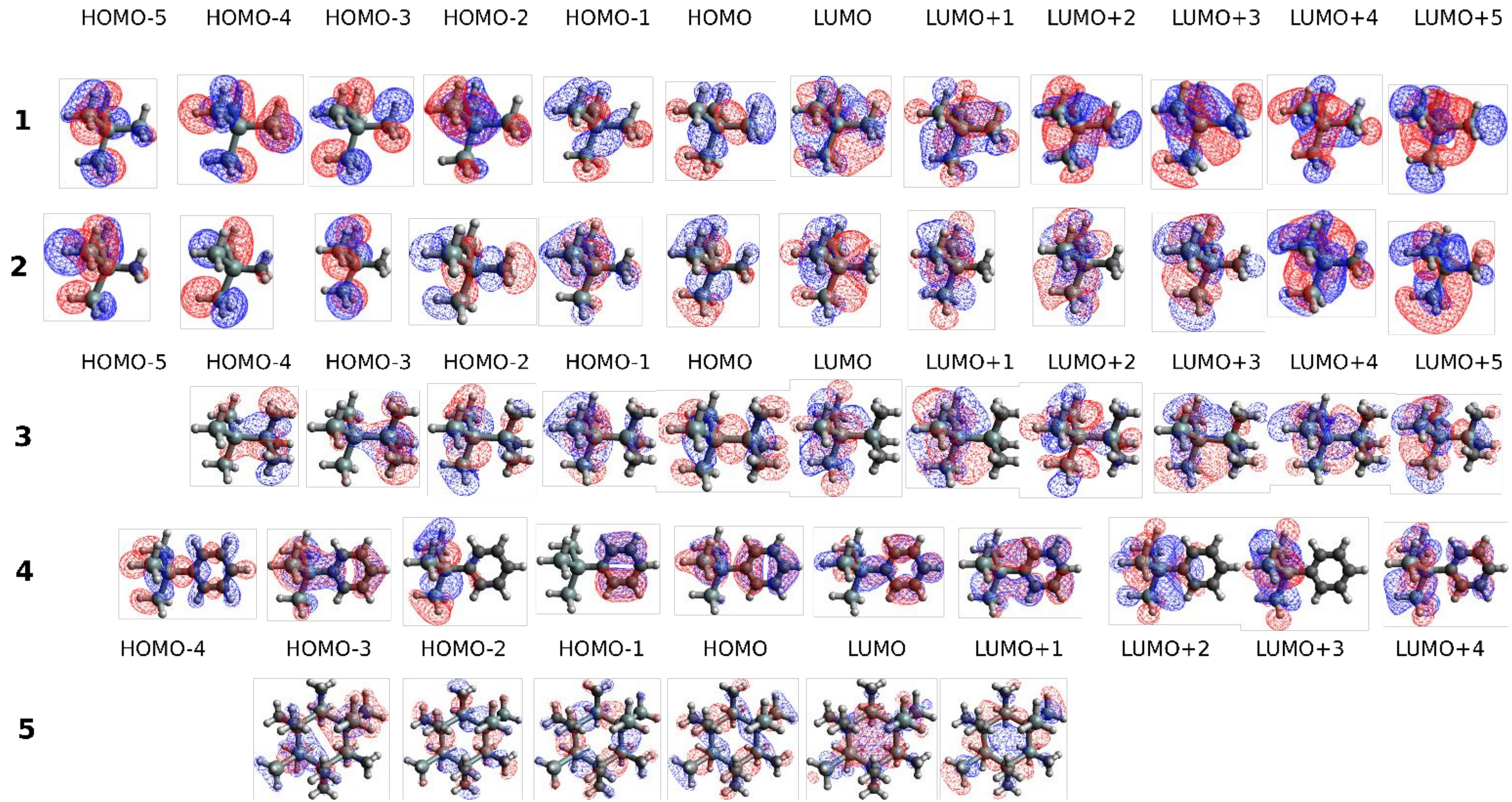

Figure S40. Molecular orbitals of compounds **1-5**, drawn with contour values of 0.05 a.u.

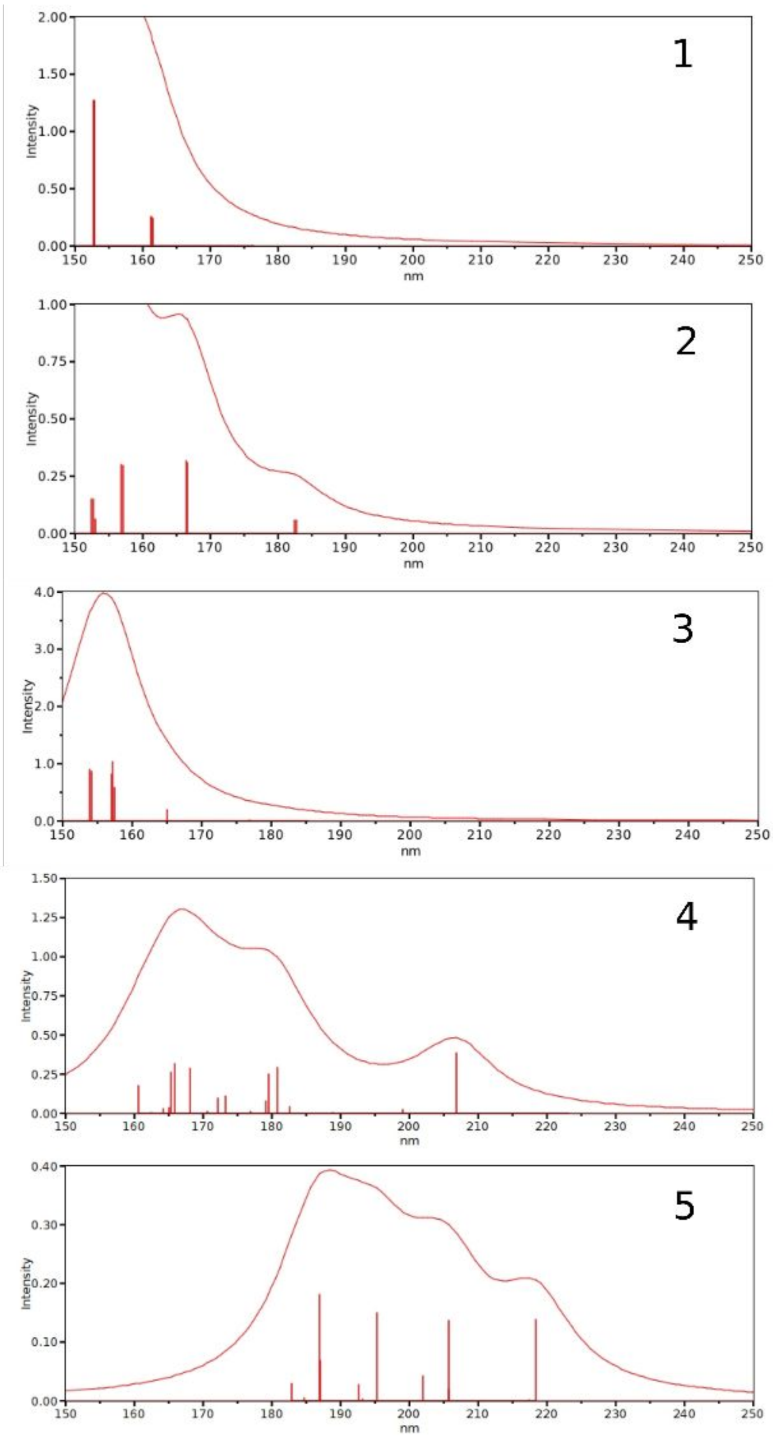

Figure S41. Simulated UV spectra of compounds **1**-**5** (from top to bottom). To simulate the spectrum, 20 vertical excitations were calculated so that the bands fall off at the edge of the low wavelengths.

References

[1] S. Grimme, J. G. Brandenburg, C. Bannwarth, A. Hansen, *The Journal of Chemical Physics* **2015**, *143*, 54107.

[2] V. Barone, M. Cossi, *J. Phys. Chem. A* **1998**, *102*, 1995–2001.

[3] M. D. Hanwell, D. E. Curtis, D. C. Lonie, T. Vandermeersch, E. Zurek, G. R. Hutchison, *Journal of cheminformatics* **2012**, *4*, 17.

[4] A.-R. Allouche, *Journal of computational chemistry* **2011**, *32*, 174–182.

[5] a) F. Neese, *WIREs Comput Mol Sci* **2018**, *8*, 33; b) F. Neese, *WIREs Comput Mol Sci* **2012**, *2*, 73–78.
